# Toward Consistent Physics-Based Modeling of Local Backbone Structures and Chirality Change of Proteins in Coarse-Grained Approaches

**DOI:** 10.1021/acs.jpclett.3c01988

**Published:** 2023-10-27

**Authors:** Agnieszka G. Lipska, Adam K. Sieradzan, Sümeyye Atmaca, Cezary Czaplewski, Adam Liwo

**Affiliations:** † Centre of Informatics Tri-city Academic Supercomputer and Network (CI TASK), Gdańsk University of Technology, Fahrenheit Union of Universities in Gdańsk, ul. G. Narutowicza 11/12, 80-233 Gdańsk, Poland; ‡ Faculty of Chemistry, University of Gdańsk, Fahrenheit Union of Universities, ul. Wita Stwosza 63, 80-308 Gdańsk, Poland; ¶ Kocaeli University, Institute of Science, Umuttepe Yerleşkesi, 41001 İzmit/Kocaeli̇, Türkiye

## Abstract

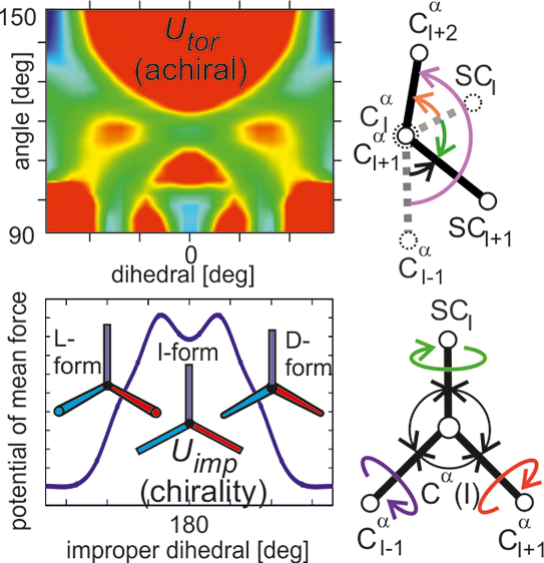

A reliable representation of local interactions is critical for the accuracy of modeling protein structure and dynamics at both the all-atom and coarse-grained levels. The development of local (mainly torsional) potentials was focused on careful parametrization of the predetermined (usually Fourier) formulas rather than on their physics-based derivation. In this Perspective we discuss the state-of-the-art methods for modeling local interactions, including the scale-consistent theory developed in our laboratory, which implies that the coarse-grained torsional potentials inseparably depend on the virtual-bond angles adjacent to a given dihedral and that multitorsional terms should be considered. We extend the treatment to split the residue-based torsional potentials into the site-based regular and improper torsional potentials. These considerations are illustrated with the revised torsional potentials and improper-torsional potentials involving the l-alanine residue and the improper-torsional potential corresponding to serine-residue enantiomerization. Applications of the new approach in coarse-grained modeling and revising all-atom force fields are discussed.

Local interactions, along with the long-range side chain–side chain and hydrogen-bonding interactions, are one of the key factors determining protein structure and dynamics.^1^ Backbone-local interactions are very likely to presculpt the protein free-energy landscape to favor regular helix and sheet structures.^2^ Therefore, the development of local-interaction-energy terms in both all-atom^3−15^ and coarse-grained^16−25^ force fields has received a lot of attention. The torsional and improper-torsional terms that describe the energy of rotation about a bond (in all-atom models) or about a virtual bond (in coarse-grained models) or the geometry of the surrounding of a central atom or site, respectively, are particularly important because a dihedral angle is a collective reaction coordinate that describes the concerted motion of four atoms or sites. It is usually assumed that the torsional terms are Fourier expressions depending only on the dihedral angles. Substantial work has been devoted to refined parametrization, in both the all-atom^10−12,15^ and coarse-grained models,^18,19,21,22^ rather than to extending the expressions to include the cross-terms dependent on planar and dihedral angles or to double/multitorsional terms.

In the all-atom representation, the expressions that involve the cross-terms depending on the valence and dihedral angles appear only in the class II force fields.^6^ The double- and multitorsional terms are even less common. Only recently the terms dependent on two consecutive torsional angles have been introduced in all-atom force fields.^13^ Earlier grid-based corrections to the peptide-backbone energy expressed in the ϕ and ψ backbone angles, which can be considered as tabulated double-torsional potentials, have been introduced to the CHARMM force field by MacKerell and colleagues (the CMAP approach).^26^ These corrections significantly improved backbone-modeling accuracy, which was reflected by a better agreement of the calculated order parameters with those determined by Nuclear Magnetic Resonance (NMR) experiments.^8^

The improper-torsional potentials consist of Fourier-type terms similar to those of the regular torsional potentials^3,4^ or are represented by the harmonic terms in the improper-dihedral^8^ or out-of-plane angles.^9^ In the all-atom force fields they are limited to the description of the deviation of the trigonal environment from planarity, while in united-atom^3^ or coarse-grained force fields^23^ they also keep the chirality of the central atom or site, respectively.

## Torsional Terms Imported from All-Atom Force Fields Are Insufficient in Coarse-Grained Approaches

In the all-atom representation, the bond angles only oscillate about the equilibrium values, and consequently, the absence of the cross-terms dependent on both the valence and dihedral angles in the torsional potentials does not seem to cause major problems with force-field accuracy. However, the backbone (C^α^···C^α^···C^α^) virtual-bond-angles in proteins alone can vary from about 75° to about 145°.^27^ Consequently, the absence of cross-terms in the torsional potentials in coarse-grained force fields could lead to major problems.

**Figure 1:**
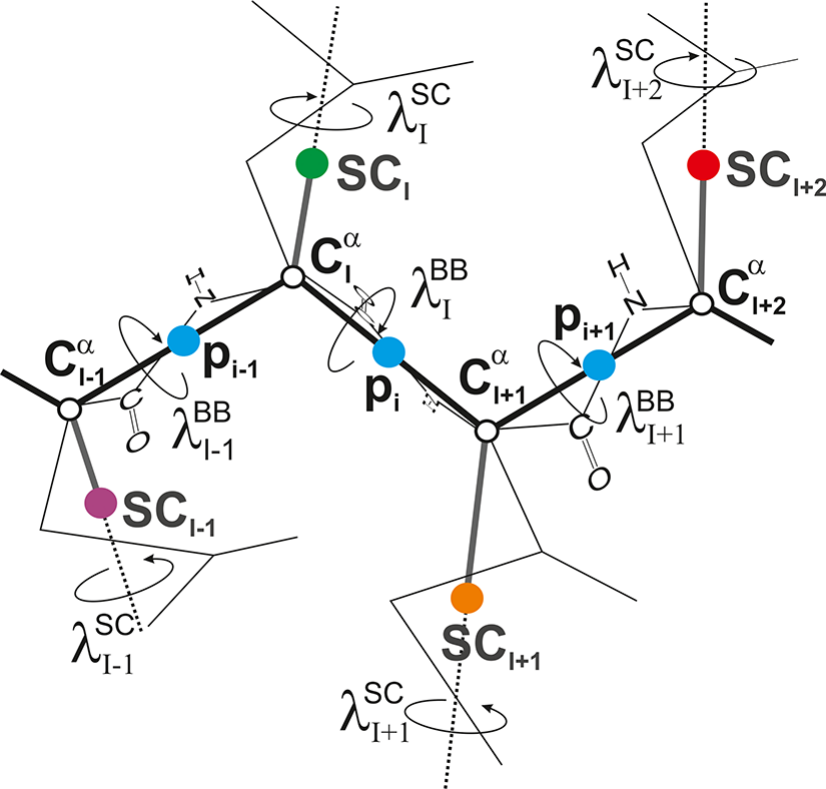
Illustration of the UNRES coarse-graining scheme with a fragment of polypeptide chain from residue *I* – 1 through *I* + 2. The interaction sites are united peptide groups (p), each located halfway between the two consecutive C^α^ atoms, and united side chains (SC), each attached to the corresponding C^α^ atom. The C^α^ atoms are not interaction sites. The geometry of the coarse-grained chain is defined by C^α^ and SC coordinates. All peptide groups are assumed in the *trans* configuration, implying the C^α^···C^α^ virtual-bond length of about 3.8 Å. The potential of mean force (PMF) of the chain is obtained by integrating the Boltzmann factors in the angles λ_*I*_^BB^, *I* = 1, 2, ..., *n*–1 and λ_*I*_^SC^, *I* = 2, 3, ..., *n*–1, *n* being the number of residues, which are the angles for the rotation of the peptide and the side chain groups (whose chemical bonds are shown as thin lines) about the respective C^α^···C^α^ or C^α^···SC virtual-bond axes, and over the solvent degrees of freedom (not shown). Residues 1 and *n* are the N- and the C-terminal blocking groups (which can be dummy groups) and do not have side chains. It should be noted that the C^α^–H^α^ groups are parts of the respective side chains, and therefore, rotation by λ_*I*_^SC^ changes the geometry of the tetrahedral surrounding of C_*I*_^α^, thus enabling us to model chirality change.

**Figure 2:**
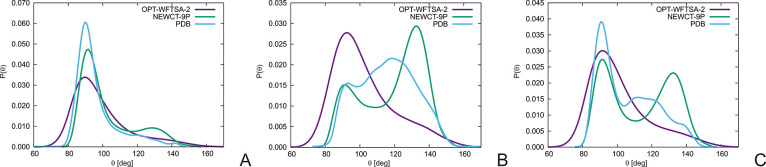
Distributions of the C^α^···C^α^···C^α^ virtual-bond angles (θ) calculated from the family of structures containing the best models of the validation-set proteins of ref (33) for the α (A), β (B), and α + β (C) proteins. Purple lines: distributions derived from the best models obtained with the OPT-WFTSA-2 variant of UNRES,^32^ in which the torsional potentials depend solely on virtual-bond-dihedral angles. Green lines: distributions derived from the best models obtained with the scale-consistent NEWCT-9P variant of UNRES,^33^ in which the torsional and correlation potentials depend on backbone-virtual-bond-dihedral and backbone-virtual-bond angles. Blue lines: distributions derived from the experimental structures. As can be seen from the panels, the distributions obtained with the OPT-WFTSA-2 force field do not exhibit any maxima at large values of virtual-bond angles θ, which are characteristic of extended structures^27^ and extend to small θ, while those obtained with the scale-consistent NEWCT-9P variant of UNRES are more similar to the distributions from the experimental structures. Adapted with permission from ref (33). Copyright 2001 AIP Publishing LLC.

At the early stages of the development of our UNited RESidue (UNRES) model for proteins (Figure 1),^28^ which is briefly described in “UNRES Model of Polypeptide Chains and Energy Function” in the Supporting Information, we were unable to parametrize a single variant of the UNRES force field that could model the structures of the α, β, and α + β proteins in the *ab initio* mode, i.e., without introducing restraints on secondary structure from knowledge-based methods or from the experiment.^29^ Different variants had to be parametrized for each secondary-structure class.^29^ This problem might also be one of the reasons that MARTINI,^18^ which is the most widely used coarse-grained force field, and many other “pragmatic” coarse-grained protein models reviewed in a recent paper^25^ can treat proteins only if secondary-structure restraints are imposed. Later, by introducing additional terms, such as the double-torsional potentials^30^ and supplemental torsional potentials involving united side chains,^31^ as well as by using the maximum-likelihood method of force-field calibration, we managed to develop a variant of UNRES good for *ab initio* modeling of proteins with any secondary-structure class.^32^ However, the modeled β-strands were too compressed, with backbone-virtual-bond angles of about 100° (Figure 2), with this feature considerably impairing the accuracy of modeling.

## Scale-Consistent Approach Embeds Atomic Details in Coarse-Grained Energy Functions

Quite recently, we developed the scale-consistent approach to the derivation of physics-based coarse-grained energy functions.^24,34^ As in our earlier work^35^ and in other physics-based approaches to coarse-graining,^36^ the prototype of an effective energy function is the potential of mean force (PMF) of a system, in which the degrees of freedom not considered in the model (collected in the vector **y**) have been integrated out, while those defining the geometry of the coarse-grained model (collected in the vector **X**) are kept, as given by eq 1.
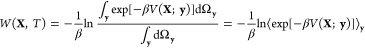
1where *W*(**X**) is the PMF, *V* is the potential energy, β = 1/*RT*, *R* is the universal gas constant, *T* is the absolute temperature, dΩ_**y**_ is the element of the volume element, and ⟨···⟩_**y**_ denotes the average of the quantity inside the brackets over **y**. The PMF is then rewritten as a sum of Kubo cluster-cumulant functions^37^ corresponding to fragments of the system, starting from the smallest and ending at the whole system; these terms have been given the name *PMF factors*.^35^ The factors corresponding to single sites or pairs of interacting sites are the first-order factors, while those corresponding to clusters of sites are higher-order terms and are not zero if the respective sites share some of the secondary degrees of freedom **y**.^35^ The expansion is cut to retain the lower-order factors; for proteins, keeping the factors up to the third order is sufficient.^34,35^ Each factor is subsequently expanded into the generalized-cumulant series, of which the first nonvanishing term is retained.^35^ It should be noted that *W* depends on the temperature; this temperature dependence is included in UNRES.

Proteins and many other biological macromolecules are linear polymers. In the UNRES model, a polypeptide chain is coarse-grained to two sites per residue, namely, the united peptide group (p, comprising the carbonyl carbon, carbonyl oxygen, amide nitrogen, and amide hydrogen for nonproline residues atoms) and the united side chain (SC, comprising the side-chain atoms and the C^α^–H^α^ group), as shown in Figure 1. Additionally, the C^α^ atoms serve as reference points and their Cartesian coordinates, along with the Cartesian coordinates of the side chain centers, are the coarse-grained variables contained in the vector **X**. The secondary variables (contained in vector **y**) are the degrees of freedom orthogonal to **X**. These are the solvent degrees of freedom and the coordinates of protein atoms constrained to a given coarse-grained geometry. Due to valence-geometry constraints, the latter can be split into the torsional angles λ^BB^ and λ^SC^ for the collective rotation of the atoms of the p and SC sites about the respective C^α^···C^α^ or C^α^···SC virtual-bond axes (Figure 1) and those corresponding to perturbations of the internal site geometry. The rotations about the virtual-bond axes will result in major changes of the distances between the atoms of different sites. In the scale-consistent approach,^24,34^ we take advantage of the fact that, at the all-atom level, the energy of a molecular system (which also includes solvent molecules) in the absence of external potential fields is a function of interatomic distances. We thus express the energy in the squares of interatomic distances (which can be done because the distances are non-negative). The squares of the distances can, in turn, be expressed in terms of the distance between the site centers, the orientation of virtual-bond axes, and the torsional angles λ,^24,34^ as outlined by eqs 2 and 3.
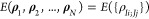
2
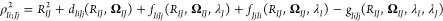
3where **ρ**_1_, **ρ**_2_, ..., **ρ**_*N*_ denote the Cartesian coordinates of the atoms that belong to site 1 through *N*, {ρ_*Ii*;*Jj*_} denotes the set of all distances between the atoms of site *I* and site *J*; *Ii* and *Jj* index the atoms of the respective sites; *R*_*IJ*_ is the distance between the centers of the sites; **Ω**_*IJ*_ denotes the variables that describe the relative orientation of sites *I* and *J*; and the functions *d*_*IiJj*_, *f*_*IiJj*_, and *g*_*IiJj*_ are defined by eqs 32–35 of ref (34) and are also recalled in the Supporting Information. The energy can be expanded in a Taylor series about the positions of coarse-grained centers, and the terms of this expansion depend on the sines and cosines of the consecutive λ angles.^34,35^ Therefore, analytical formulas for the cumulant terms are obtained by integrating the trigonometric functions of the λ’s; the coupling terms arise when the products of *f*’s or *g*’s that share at least one λ angle are integrated.^34,35^ The obtained analytical expressions depend on the orientation of the sites involved and thus reflect their atomic-scale details; hence, the approach has been termed the scale-consistent approach. Details of the approach can be found in refs (24) and (34).

## Torsional Potentials Exhibit Cross-Dependence on Adjacent Virtual-Bond Angles

The torsional potentials are second-order terms that pertain to the coupling of the interactions between consecutive units. It has been a common practice, which started from the seminal work by Levitt,^16^ to extend the local interactions contained in the torsional potential beyond interacting sites. For the coarse-grained models of proteins this means that a unit includes the C^β^ atom for nonglycine residues and even all proline side-chain atoms for proline, due to the cyclization of the side proline chain with the backbone.^16^ This description has been applied in the UNRES model, which consequently has three types of residues with regard to the torsional and other local potentials: glycine, alanine, and proline. With *e*_*I*_ and *e*_*I*+1_ denoting the local energy surfaces of units *I* and *I* + 1, the torsional contribution to the potential of mean force is given by eq 4 (see also eq 68 in ref (34)).
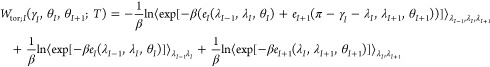
4

**Figure 3:**
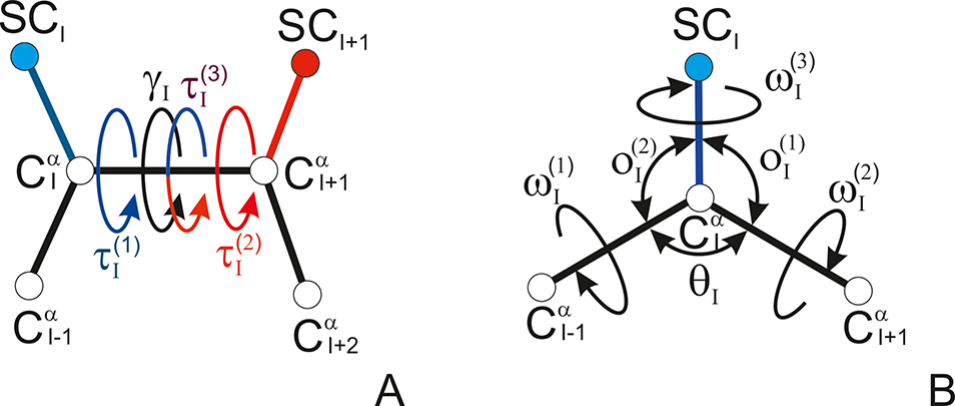
(A) Illustration of the backbone-dihedral angle γ_*I*_ and the dihedral angles τ_*I*_^(1)^, τ_*I*_^(2)^, and τ_*I*_^(3)^ involving the side chains in the UNRES model of polypeptide chains. These dihedral angles are defined as the angles for counterclockwise rotation of the plane defined by C_*I*_^α^, C_*I*+1_^α^, and Y from that defined by X, C_*I*_^α^, C_*I*+1_^α^ about the C_*I*_^α^··· C_*I*+1_^α^ axis when looking from C_*I*+1_^α^ to C_*I*_^α^, X = C_*I*–1_^α^, Y = C_*I*+2_^α^ for γ_*I*_, X = SC_*I*_, Y = C_*I*+2_^α^ for τ_*I*_^(1)^, X = C_*I*–1_^α^, Y = SC_*I*+1_ for τ_*I*_^(2)^ and X = SC_*I*_, Y = SC_*I*+1_ for τ_*I*_^(3)^. For better illustrations the curved arrows are colored after SC_*I*_ and SC_*I*+1_ for τ_*I*_^(1)^ and τ_*I*_^(2)^, respectively and the curved arrow is two-colored for τ_*I*_^(3)^. (B) Illustration of the planar angles θ_*I*_, *o*_*I*_^(1)^, and *o*_*I*_^(2)^ and the improper-torsional angles ω^(1)^, ω^(2)^, and ω^(3)^. The improper dihedral angles are the dihedral angles for the counterclockwise rotation of the of the plane defined by Z, C^α^_i_, X from that defined by Y, C_*I*_^α^, X about the C_*I*_^α^···X virtual-bond axis when looking from C_*I*_^α^ toward X, where X = C_*I*–1_^α^, Y = C_*I*+1_^α^, Z = SC_*I*_ for ω_*I*_^(1)^; X = C_*I*+1_^α^, Y = SC_*I*_, Z = C_*I*–1_^α^ for ω_*I*_^(2)^; and X = SC, Y = C_*I*–1_^α^, Z = C_*I*+1_^α^ for ω_*I*_^(3)^.

The resulting expression depends on the virtual-bond-dihedral angle γ_*I*_ and the virtual-bond-angles θ_*I*_ and θ_*I*+1_ (Figure 3) and not just on γ_*I*_ as in the other coarse-grained force fields and in earlier versions of UNRES. The dependence on γ_*I*_ arises from the fact that the energy surfaces of the *I*th and the *I* + 1th residue share the same torsional angle λ_*I*_ to be averaged over, which has to be shifted when passing to the local coordinate system of the neighboring residue.^27,34,35^ The averaging over the angles λ for the rotation about virtual-bond axes implies cylindrical symmetry of the mean-field local interactions and is very similar in spirit to the recent model of backbone interactions in proteins developed by Banavar and co-workers,^38^ in which the interaction sites are represented by coin-like sites threaded on a chain. Such sites have axial symmetry, which also arises when averaging the Boltzmann factors in eq 4 over angles λ.

To the second-order approximation (implemented in the present UNRES), the scale-consistent torsional potentials can be expressed by eq 5.^34^ This expression originates from averaging of various powers of the *f* contributions to the square of the interatomic distances (expressed by eq 3) between unit *I* and unit *I* + 1 and those between units *I* + 1 and *I* + 2 and can thus be shorthanded as ⟨*ff*⟩.^34^
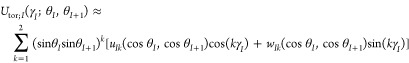
5where *u*_*i*_ and *w*_*i*_ are polynomials in cos θ_*I*_ and cos θ_*I*+1_ whose coefficients depend on the types of pairs of connected sites, i.e., on the number of residue kinds. Therefore, the number of parameters grows only linearly with the number of residue types.^33,34^ Moreover, it is sufficient to change the sign of some of the parameters if a residue changes its chirality, which enables us to develop the parameters dependent only on the kind and not on both the kind and chirality of a residue.^33,34^

The most important feature of the torsional potential defined by eq 5 is that the terms with an γ-angle multiplicity of *k* are multiplied by the *k*th power of the product of the sines of the adjacent θ angles. This is the dominant part of the simultaneous dependence of the torsional potentials on both the virtual-bond-dihedral angles and virtual-bond angles. Its immediate consequence is that, when a chain fragment is nearly linear, the torsional potential tends to 0. Without this dependence, the torsional potentials are undefined for linear chain fragments and numerically unstable for nearly linear fragments. Based on numerical-stability considerations, Bulacu et al.^20^ proposed a similar form of a coupled angular and torsional potential, albeit with the fixed power of 3 of the sin θ_*I*_ sin θ_*I*+1_ term.

Because the weights of the terms with different γ-angle multiplicity strongly depend on the θ angles, which are close to 90° for helices and turns and more open for extended chains that constitute β-structures,^27^ the torsional potentials are very different for the helical and β-sheet sections of protein structure. Likewise, the dependence on the virtual-bond angles has been introduced to the correlation terms that couple the backbone-local and backbone-electrostatic interaction in UNRES.^34^ The modified potentials were parametrized based on *ab initio* energy surfaces of model systems, and the new UNRES force field was calibrated by using the maximum-likelihood method.^33^ The modified UNRES is able to model the structures of proteins of any secondary-structure class, as tested with benchmark proteins^33^ and, subsequently,^39^ in the 13th Community Wide Experiment on the Critical Assessment of Techniques for Protein Structure Prediction, CASP13.^40^ As opposed to the previous variants of UNRES, the virtual-bond-angle distribution calculated from the modeled structures of the β- and α + β-proteins are bimodal, with the lobe centered at more open angles corresponding to the extended sections of the chain (Figure 2).^33^

In our recent work we explored the coupling of local conformational states along polypeptide chains.^41^ We derived analytical formulas for the torsional potentials for backbone segments of arbitrary length. The general formulas contain the cosine terms dependent on combinations of the consecutive virtual-bond-backbone dihedrals with both plus and minus signs. However, for the specific cases of extended strands, as encountered in β-sheets, the dominant term depends on the sum of consecutive dihedral angles. Consequently, a concerted rotation of the segment as a whole, as a result of which the rotation of one end reciprocated by the rotation of the other end is accomplished at reduced free-energy cost (similar to turning a key in the lock).^41^ For segments with the virtual-bond angles θ close to 90° as in helical structures, we found that there is a multitorsional term dependent on the product of phase-shifted cosines of the consecutive backbone dihedrals that stabilizes the chain conformation at either end of a helix. We proposed the respective multitorsional-potential formulas for coarse-grained force fields.^41^

## Residue-Based Torsional Potentials Incorporate Regular and Improper Torsional Terms

The residue-based torsional and multitorsional potentials described above pertain to backbone dihedral angles. Selecting those only to describe torsional-energy surfaces implies that the torsional potentials are averaged over the positions of united side chains corresponding to a given geometry of the respective backbone segment. In the UNRES model, this approach implies that the torsional part of the effective energy function is more coarse-grained than the other ones. Moreover, the torsional potentials partially overlap with the side-chain-rotamer potentials (*U*_rot_).^42^ Introduction of the torsional potentials that correspond to the rotation of the interaction sites defined in the model about a given backbone virtual bond results in a much clearer separation of effective energy contributions. Moreover, because the united side chains in UNRES are quite flexible with respect to the backbone,^42^ the torsional angles with a common virtual-bond are not as strictly related to each other as, e.g., the torsional angles for the rotation of the hydrogen atoms of a methyl group about a C–X bond.

The potentials dependent on the distance between the side chains of the neighboring residues, which is related to the cosine of the τ^(3)^ dihedral angle defined in Figure 3A, as well as between those of up to fourth-neighbor residues, were first introduced by Koliński^17^ in the statistical C^α^, C^β^, and Side chain (CABS) force field. In our earlier work^31^ we introduced supplemental torsional potentials dependent on the virtual-bond angles τ^(1)^, τ^(2)^, and τ^(3)^ shown in Figure 3A. These potentials improved the performance of UNRES,^31,43^ but because they contained 400 sets of parameters (the number of pairs of types of consecutive amino-acid-residues), they were difficult to refine.

In the present UNRES force field there are no explicit improper-torsional potentials to account for the energetics of the rotamers of the united side chains. The energetics of side-chain rotamers is represented by the local side-chain energy terms, *U*_rot_.^42^ For the residue with index *I*, *U*_rot_ depends explicitly on the coordinates of SC_*I*_ in the local coordinate system of this residue and on the backbone virtual-bond angle θ_*I*_. The respective formulas have been developed^42^ to obtain a good fit to the respective potentials of mean force calculated for terminally blocked amino-acid residues by means of the semiempirical PM3 method^44^ of molecular quantum mechanics. In this Perspective, we propose the site-based local-interaction-energy terms that can be used in UNRES and in other coarse-grained force models for (bio)polymer systems. The local site–site-interaction energy can be expressed by eq 6 (it should be noted that the virtual-bond-deformation contributions are not included because they pertain to intrasite interactions).
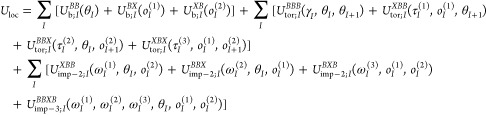
6where the subscripts b, tor, and imp denote the virtual-bond-angle deformation (“bending”), virtual-bond-torsional and virtual-bond-improper-torsional terms, respectively, and *B* and *X* in superscript denote a backbone or a side chain site, respectively. Their appearance in superscripts indicates the types of sites involved in the respective terms. The virtual-bond angles θ, *o*^(1)^, and *o*^(2)^, the virtual-bond-dihedral angles γ_*I*_ and τ_*I*_^(*m*)^,*m* = 1,2,3, as well as the virtual-bond improper dihedral angles ω_*I*_^(*m*)^;*m* = 1,2,3 are shown in Figure 3. It should be noted that, for clarity, we omit from eq 6 the energy-term weights and the temperature factors that scale the higher-order cumulant terms of UNRES to account for the fact that the UNRES energy function has the sense of free energy.^24^

The virtual-bond-angle-bending potentials, *U*_b_, correspond to the first-order PMF factors, the regular torsional potentials are second-order PMF factors, which correspond to the coupling of the local interactions between two pairs of neighboring sites sharing a common C^α^··· C^α^ virtual bond, while the three improper torsionals with superscripts denoting three groups are the second-order PMF factors corresponding to the coupling between the local interactions of the first and the last site with the middle site. The last improper-torsional term, *U*_imp;*I*_^BBXB^ is the third-order factor in the PMF, which corresponds to the coupling of the local interactions between the three pairs of UNRES sites (two backbone groups and one side chain) attached to a common C^α^ atoms. There are no other third-order nonzero PMF factors. Yet higher-order terms, dependent on both regular and improper dihedrals, could be introduced, but those mentioned above seem to account for the energetics of local interactions at the coarse-grained level. Thus, the former residue-based torsional potentials corresponding to a backbone segment of four consecutive C^α^ atoms are split into four backbone torsional potentials and two sets of improper-torsional potentials dependent on the planar, dihedral, and improper-dihedral angles shown in Figure 3.

The backbone-only virtual-bond-angle and virtual-bond-torsional potentials, *U*_b_^BB^ and *U*_tor_^BBB^, are equivalent to those determined in our earlier work for the glycine residue or a pair of consecutive glycine residues, respectively.^33,34^ However, the residue-based backbone-virtual-bond-bending potentials for the other residues in UNRES contain contributions from the *U*_b_^BX^, *U*_b_^XB^ virtual-bond-bending potentials, and average contributions from improper-torsional potentials. The latter pertain to the local side-chain potentials and, consequently, overlap with the torsional potentials if those are controlled only by the backbone variables.

A characteristic feature of the torsional potentials present in eq 6 is that each of them is achiral (see “Torsional and Improper-Torsional Potentials of Order 2” in the Supporting Information). They can produce a residue-based chiral potential only when put together given the chirality of residues *I* and *I* + 1. The symmetry of the torsional potentials results from the fact that the peptide group is planar, and therefore, the energy surfaces of systems composed of two interacting peptide groups or a peptide group interacting with a side chain are symmetric. To the second-order approximation, the formulas for the site-based torsional potentials are thus expressed by eqs 7–10, respectively.
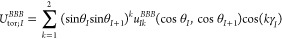
7
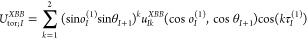
8
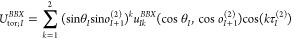
9
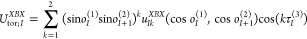
10where the planar and dihedral angles are shown in Figure 3A. The parameters of the torsional potentials can be expressed in terms of quantities dependent on single site type.

The expressions for the improper-torsional potentials, which arise from second-order PMF factors are similar (eqs 11–13) except that *U*_imp–2;*I*_^BXB^ includes a phase shift if the respective side chain is intrinsically chiral (see “Torsional and Improper-Torsional Potentials of Order 2” in the Supporting Information).
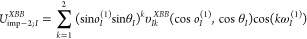
11
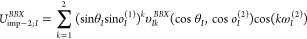
12
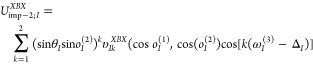
13where the phase angle Δ_*I*_ is not zero only for isoleucine and threonine, the side chains of which are intrinsically chiral, and the symbol υ is used to denote the polynomial in the cosines of the two adjacent planar angles to distinguish it from that in the regular torsional potentials (eqs 7–10).

The torsional contribution to the effective energy corresponding to the third-order PMF factor consists of the ⟨*fgf*⟩ and the ⟨*ggg*⟩ averages and is expressed by eq 14 (see “Improper Torsional Potentials of Order 3” in the Supporting Information for derivation). For clarity, we keep only the first term arising from the generalized cumulant expansion. In practical implementation, it can be expanded to include the torsional terms with a higher multiplicity and the complementary powers of the terms with virtual-bond angles.
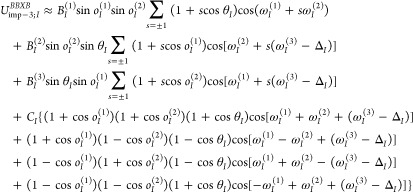
14where *B*_*I*_^(1)^–*B*_*I*_^(3)^ and *C*_*I*_ are adjustable parameters and Δ_*I*_ is not zero only for isoleucine and threonine.

**Figure 4:**
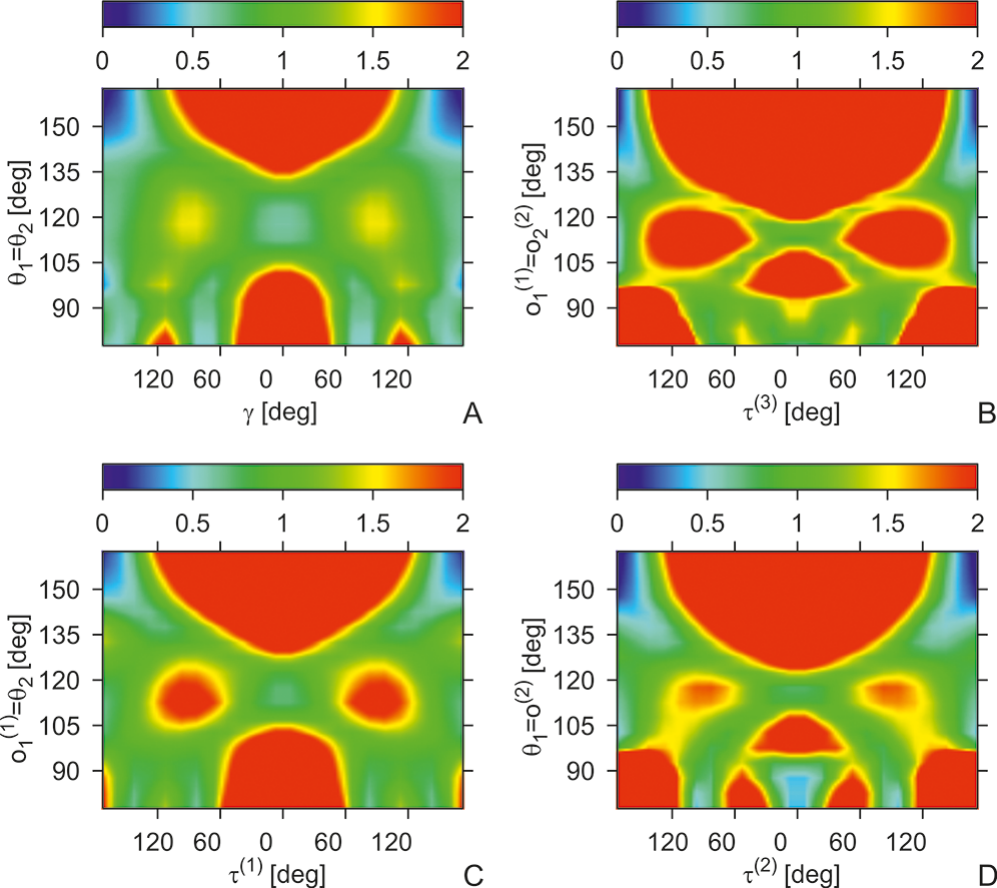
Heat maps (color scale in kcal/mol) of the *U*_tor_^BBB^(γ,θ_1_,θ_2_) (A), *U*_tor_^XBX^(τ^(3)^,*o*_1_^(1)^,*o*_2_^(2)^) (B), *U*_tor_^XBB^(τ^(1)^,*o*_1_^(1)^,θ_2_) (C), and *U*_tor_^BBX^(θ_1_,τ^(2)^,*o*_2_^(2)^) (D) torsional potentials involving the backbone and the side chains of the Ala-Ala system. The potential-energy surfaces were calculated with the PM7 semiempirical method^45^ with the PCM solvation model.^46^ The drawings were made with gri.^47^

Based on the energy surfaces of terminally blocked glycine (Ac-Gly-NHMe) and incomplete terminally blocked amino-acid residues Ac-NH-C^α^-Me or Me-C^α^H_2_-CO-NHMe calculated with the semiempirical PM7 method of molecular quantum mechanics^45^ with the Polarizable Continuum Model (PCM) of solvation,^46^ we determined the potentials of mean force corresponding to the angle, torsional, and second-order improper-torsional terms in eq 6 for the alanine residue (see “Calculation of the torsional and improper-torsional PMF surfaces” of the Supporting Information). The selection of a relatively inexpensive semiempirical method was motivated by the necessity of doing the calculations for all 20 types of natural amino-acid residues. The results for other residue types as well as parametrization of the potentials and their implementation in UNRES will be published elsewhere. The heat maps of the torsional PMFs are shown in Figure 4. Consistent with eq 7, the maps are symmetric at the γ and τ angles.

**Figure 5:**
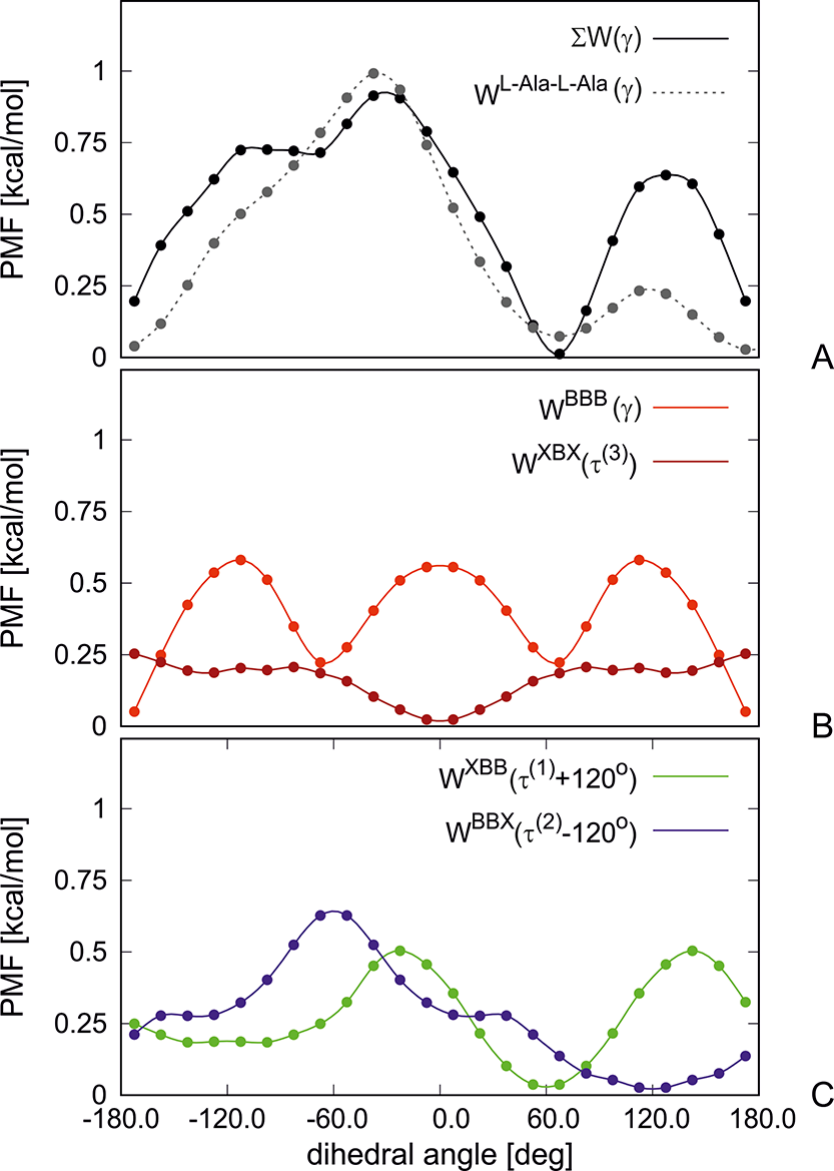
Torsional potentials of the l-Ala-l-Ala system averaged over the virtual-bond angles (B, C) and their sum compared with the total torsional potential calculated by integrating over the potential-energy maps of Ac-l-Ala-NHMe (A). The PM7 semiempirical method^45^ with the PCM implicit solvation model^46^ was used. To add the potentials in panel A and to make them the function of the backbone virtual-bond dihedral angle γ it was assumed that τ^(1)^ = γ + 120° and τ^(2)^ = γ – 120°, corresponding to average phase shifts for the l-alanine residue. The drawings were made with gnuplot.^48^

Assuming that residue chiralities are fixed, we can understand how they translate to the asymmetry of the torsional potentials that are used in the present UNRES,^33,34^ which were calculated based on the energy surfaces of whole terminally blocked residues. If both adjacent residues have the L-chirality, the virtual-bond-dihedral angles τ^(1)^, τ^(2)^, and τ^(3)^ involving the side chains are correlated with the backbone-virtual-dihedral-dihedral angle γ_*I*_ so that τ_*I*_^(1)^ ≈ γ + 120°, τ_*I*_^(2)^ ≈ γ – 120°, and τ_*I*_^(3)^ ≈ γ_*I*_. In Figure 5 the four constituent potentials and their sum are plotted after implementing the phase shift of τ^(1)^ and τ^(2)^ and after Boltzmann averaging over the virtual-bond angles θ, *o*^(1)^, and *o*^(2)^. It can be seen that the sum of the four potentials has deep minima at γ = 60° and γ = 180° corresponding to the right-handed helix and extended structure, respectively, and the global maximum at γ = −30°. A shallow minimum at γ = −60°, corresponding to the left-handed helix, also appears. Inspection of the panels of Figure 5 enables us to conclude that the torsional potential resulting from the coupling of the Ac-NH-C^α^H_2_-Me and Ac-Gly-NHMe energy surfaces (which is shifted by +120°) outweighs that of the Me-C^α^H_2_-CO-NHMe (which is shifted by −120°), and consequently, the sum of the four torsional potentials exhibits asymmetry.

On the other hand, the minima corresponding to helical structures are too deep compared to those in the PMF calculated from the complete energy surfaces of two terminally blocked alanine residues, which is also shown in the upper panel of Figure 5. This means that higher-order terms in the cluster-cumulant expansion make a significant contribution to the local potentials. These contributions are implicitly taken into account while calculating the torsional PMF surfaces from the energy surfaces of terminally blocked amino-acid residues. The most viable contributions to the “bulk” backbone-torsional potentials used in state-of-the-art UNRES are the improper-torsional potentials, which consist of second- and third-order contributions.

## Scale-Consistent Improper-Torsional Terms Describe Enantiomerization

**Figure 6:**
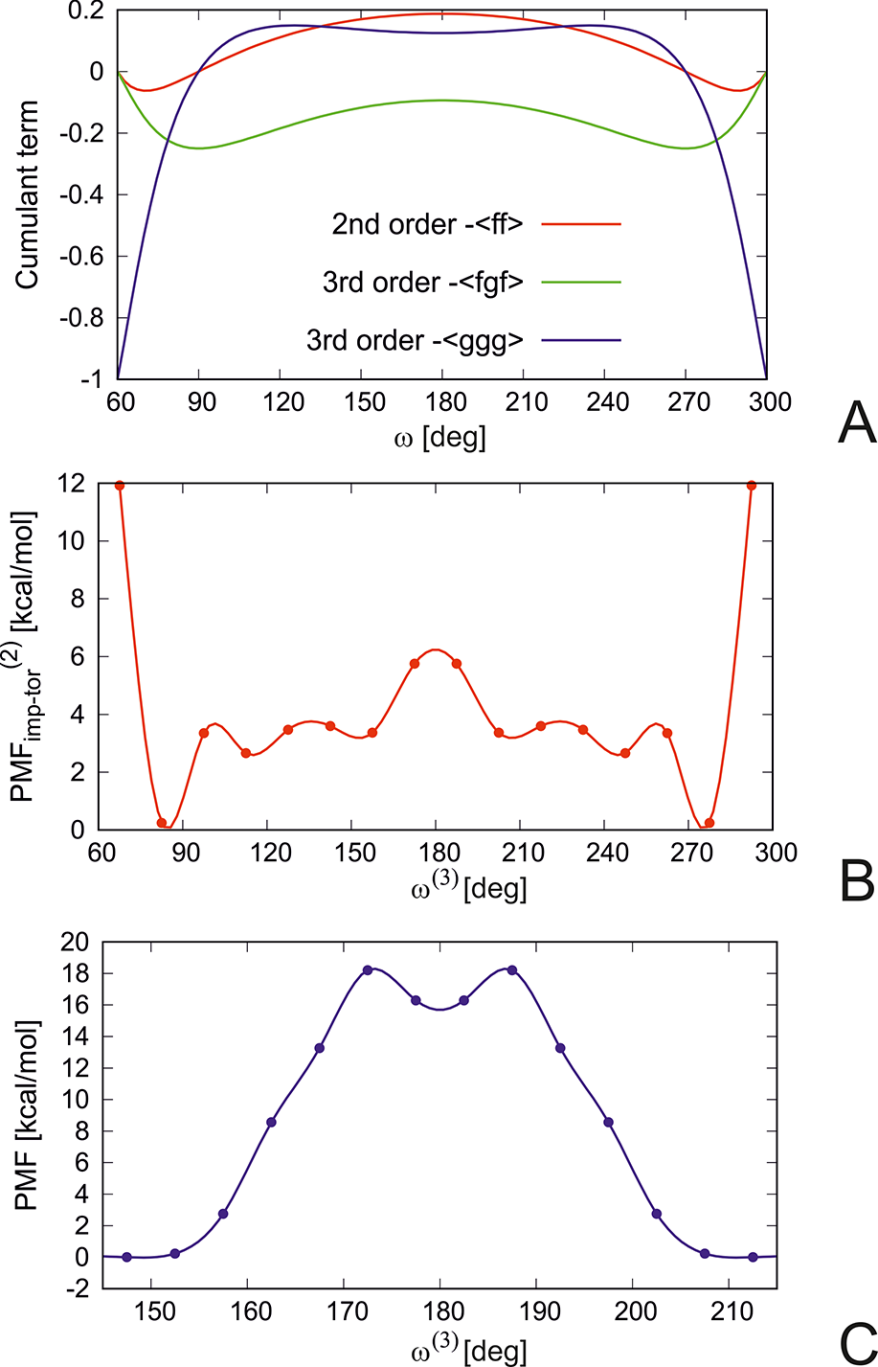
Profiles of (A) the lowest-order cluster-cumulant approximations to second-order (eqs 11–13) and third-order torsional potentials (eq 14); (B) the second-order improper-torsion PMF of terminally blocked alanine residue determined by Boltzmann integration of the potential-energy surfaces of Ac-Gly-NHMe, Me-CH_2_-CONHMe, and Ac-NH-CH_2_-Me semiempirical method of molecular quantum mechanics with the PCM solvation model; and (C) the enantiomerization pathway of a terminally blocked serine residue calculated by the PM7 semiempirical method^45^ with the PCM solvation model.^46^ In panels A and B all improper torsional angles are equal to each other, ω^(1)^ = ω^(2)^ = ω^(3)^, which implies that the planar angles θ, *o*^(1)^, and *o*^(2)^ are determined. The drawings were made with gnuplot.^48^

The second- and third-order cluster-cumulant contributions to the improper-torsional potentials are plotted in Figure 6A (assuming that all three improper-torsional angles ascribed to a C^α^ point are equal). It can be seen that the second-order contribution has a maximum at ω = 180° and shallow minima at ω = ± 70° (or ω = 70° and 290° in Figure 6, in which the angle ranges from 0° to 360° to put 180° in the center), thus covering both chiralities. This profile is similar to the second-order torsional PMF calculated from the potential-energy surfaces of Ac-Gly-NHMe, Ac-HN-CH_2_-Me, and Me-CH_2_-CO-NHMe shown in panel B of the figure, except that the latter has a finer structure. Clearly, the complete improper-torsional potential cannot be evaluated by integration over the energy surfaces of the chunks of the system sharing a common C^α^ atom because large changes of the improper torsionals that can lead to chirality change imply a chemical reaction and, thus, require a full quantum-mechanical description, which gives rise to the third-order improper-torsional terms. In fact, it can be seen from Figure 6B that although the planar structure (which can be identified with the planar intermediate in the chirality-change reaction) is 6 kcal/mol higher in terms of PMF than the PMF-minimum structures, this barrier is by far too small.

It is interesting that the ⟨*ggg*⟩ term (the green line in Figure 6A) has a minimum at ω = 180° and maxima at both sides of the minimum region. These topological features suggest that the minimum could be related to a planar (achiral) intermediate and the maxima with the transition states in the enantiomerization process. One of the mechanisms of amino-acid enantiomerization involves achiral enol intermediates.^49^ Using the PM7 semiempirical method^45^ with the PCM solvation model,^46^ we determined the water-mediated enolization-reaction pathway of the terminally blocked serine residue (see the Supporting Information) and used the obtained energy profile to calculate the respective PMF, which is shown as a function of ω^(3)^ (however, all three ω angles are very similar) in Figure 6C. It can be seen from the figure that the profile is topologically similar to that of the ⟨*ggg*⟩ term except that the maxima are closer to the enol minimum. This result suggests that our new description of coarse-grained local potentials can be applied to cover such complicated phenomena as amino-acid enantiomerization, which is implicated in a variety of diseases, including cataracts in elderly people.^50^

The partition of the local-interaction potentials given by eq 6 and the formulas for the torsional and improper-torsional potentials could also be applied to improve all-atom force fields. It has been demonstrated that the torsional potentials result from through-bond coupling of electron–electron interactions.^51^ Since the electron probability density exhibits axial symmetry about the bond axis, the averaging over electron density (which, within the Born–Oppenheimer approximation, gives the potential-energy surface of a system expressed in nuclear coordinates) is mostly done about the bond axis. Thus, the approach proposed in this work provides a systematic way for finding the cross-terms dependent on both the valence and dihedral angles. Moreover, the ⟨*ggg*⟩ contribution to the third-order improper-torsional term depends on all three improper-dihedral angles and all three planar angles defined by the three atoms attached to a common atom. Because the cosines of the improper-dihedral angles are unambiguously defined by the planar angles in such a case (see eqs S33–S35 of the Supporting Information), this term depends on the complete geometry of the surrounding of the center and will not drive at geometrically inconsistent angles and improper dihedrals. Furthermore, the generalization to coordination numbers higher than 3 is feasible. This extension could improve the modeling of metal-ion coordination at the force-field level.

## Scale-Consistent Methodology Can Facilitate Force-Field Development and Enrich Machine-Learning Approaches to Model Protein Structure and Dynamics

The methodology presented in this work provides force-field developers with ready-to-use physics-based expressions for the torsional, multitorsional, and improper-torsional potentials, as well as with the methodology to derive new expressions if needed, which is an obvious advantage over finding the formulas on a heuristic basis or directly incorporating precalculated local-interaction-energy surfaces as in the CMAP approach.^26^ Clearly, the formulas need to be parametrized considering detailed interactions in a system under study. Here the machine-learning algorithms can be helpful because they can be trained not only to find parameters of the prederived formulas but also to find more appropriate formulas based on the formalism summarized in this Perspective. Moreover, introducing the physics-based combinations of virtual-bond and virtual-bond-dihedral angles and their sequences along the chain in the machine-learning algorithms of protein-structure modeling such as AlphaFold^52^ could improve their performance in protein-structure prediction and extend their scope to modeling the conformational ensembles of intrinsically disordered proteins (IDPs), proteins with intrinsically disordered regions (IDRs), and protein dynamics. That the machine-learning algorithms benefit from introducing improved scoring or activation functions is well illustrated with examples of neural networks, which were initially based on linear activation functions. This feature halted their development in the 1970s, and they came into play again after introducing nonlinear activation functions.^53^

## Supporting Information Available

Description of the UNRES model, details of the mathematical considerations, and information on the calculations illustrating the formulas for the new torsional and improper-torsional potentials (PDF)
