# Supplementary material for: Toward Consistent Physics-Based Modeling of Local Backbone Structures and Chirality Change of Proteins in Coarse-Grained Approaches
Source: J Phys Chem Lett. 2023 Oct 27;14(44):9824–33. doi: 10.1021/acs.jpclett.3c01988 (PMC10641867; doi:10.1021/acs.jpclett.3c01988)
Supplement: Supplementary file 1 — jz3c01988_si_001.pdf [file jz3c01988_si_001.pdf]

# Toward Consistent Physics-Based Modeling of Local Backbone Structures and Chirality Change of Proteins in Coarse-Grained Approaches

Agnieszka G. Lipska,[†] Adam K. Sieradzan,[†,‡] Sümeyye Atmaca,[¶] Cezary Czaplewski,[†,‡] and Adam Liwo[*,†,‡]

†*Centre of Informatics Tri-city Academic Supercomputer and Network (CI TASK), Gdańsk University of Technology, Fahrenheit Union of Universities in Gdańsk, ul. G. Narutowicza 11/12, 80-233 Gdańsk, Poland*

‡*Faculty of Chemistry, University of Gdańsk, Fahrenheit Union of Universities, ul. Wita Stwosza 63, 80-308 Gdańsk, Poland*

¶*Kocaeli University, Institute of Science, Umuttepe Yerleşkesi, 41001, İzmit/Kocaeli, Türkiye*

E-mail: adam.liwo@ug.edu.pl

Phone: +48 58 5235124. Fax: +48 58 5235012

## UNRES model of polypeptide chains and energy function

As stated in the main text, in the UNRES model, a polypeptide chain is represented by a sequence of $\alpha$-carbon ($C^{\alpha}$) atoms linked by virtual bonds, with peptide groups (p) located halfway between the consecutive $C^{\alpha}$s and united side chains attached to the $C^{\alpha}$s with the

$C^\alpha \cdots SC$ virtual bonds (Figure 1 of the main text).[1] Only the ps and the SCs are interaction sites, while the $C^\alpha$s assist in chain-geometry definition. The sites and the corresponding interaction potentials have axial and not spherical symmetry. The solvent is implicit in the interaction potentials and the present parameterization corresponds to physiological pH and physiological salt concentration. Quite recently an extension to lipid-membrane environment was developed,[2] in which the $U_{SC_iSC_j}$, $U_{p_ip_j}$, $U^{(3)}_{corr;ij}$ and $U^{(3)}_{turn;i}$ terms were modified to account for their entering the lipid environment. The local terms were kept unchanged since they primarily depend on steric interactions. The current UNRES energy function is expressed by eq S1.

$$\begin{aligned} U &= w_{SC}\sum_{i<j} U_{SC_iSC_j} + w_{SCp}\sum_{i\neq j} U_{SC_ip_j} + w_{pp}^{VDW}\sum_{i<j-1} U_{p_ip_j}^{VDW} + w_{pp}^{el} f_2(T)\sum_{i<j-1} U_{p_ip_j}^{el} \\ &+ w_{tor} f_2(T)\sum_i U_{tor}(\gamma_i,\theta_i,\theta_{i+1}) + w_b\sum_i U_b(\theta_i) + w_{rot}\sum_i U_{rot}(\theta_i,\hat{\mathbf{r}}_{SC_i}) \\ &+ w_{bond}\sum_i U_{bond}(d_i) + w_{ssbond}\sum_i U_{ssbond}(d_i^{SS}) \\ &+ w_{corr}^{(3)} f_3(T)\sum_{i<j-1} U^{(3)}_{corr;ij} + w^{(3)}_{turn} f_3(T)\sum_i U^{(3)}_{turn;i} \end{aligned} \tag{S1}$$

where the terms $U_{SC_iSC_j}$ (with axial symmetry) are sidechain-sidechain interaction energies,[3,4] $U_{SC_ip_j}$ are excluded-volume potentials that prevent the collapse of the united sidechains on the backbone, $U_{p_ip_j}^{VDW}$ (with spherical symmetry) and $U_{p_ip_j}^{el}$ (with axial symmetry) are the non-bonded and mean-field-electrostatic interaction potentials of united peptide groups, $U_{bond}$ are the bond-deformation potentials, $U_b$ and $U_{tor}$ are the backbone-virtual-bond-angle and the backbone-virtual-bond-torsional potentials, respectively (it should be noted that the latter depend not only on the virtual-bond dihedral but also on virtual-bond angles, which feature results from the application of the scale-consistent methodology to the derivation of these potentials[5,6]), $\theta_i$ and $\gamma_i$ denoting the virtual-bond- and virtual-bond-dihedral angles, respectively (Figure 1 in the main text), $U_{rot}$ are the sidechain-rotamer potentials, in which

$\hat{\mathbf{r}}_{SC_i}$ denotes the local coordinates of the unit vector pointing from $C_i^\alpha$ to $SC_i$,[7] while $U_{corr}^{(3)}$ and $U_{turn}^{(3)}$ are multibody (correlation) terms that account for the coupling of the backbone-local and backbone-electrostatic interactions.[5,8] The correlation terms are essential in modeling regular $\alpha$-helical and $\beta$-sheet structures.[5,8,9] $U_{ssbond}$ denotes the terms that account for the energetics of disulfide bonds, including their formation and breaking.[10,11] The $w$s are the weights of the energy terms and have been determined, along with some other parameters, by maximum-likelihood calibration of the force field.[12]

The factors $f_n(T)$ account for the dependence of the force-field terms that correspond to higher-order terms in the Kubo cluster-cumulant expansion on temperature,[13] as given by eq S2.

$$f_n(T) = \frac{\ln\left[\exp(1) + \exp(-1)\right]}{\ln\left\{\exp\left[(T/T_\circ)^{n-1}\right] + \exp\left[-\left(T/T_\circ\right)^{n-1}\right]\right\}} \tag{S2}$$

where $T_\circ = 300$ K. The temperature dependence appears because UNRES originates from the potential of mean force of polypeptide chains in water, which depends on temperature.

The rigorous physics-based derivation of UNRES, which results in the dependence of interaction energies on site orientation, and the presence of physics-based multibody (correlation) terms make UNRES different from most of the other coarse-grained models, which are based on "neo-classical" expressions imported from all-atom force fields.[14] Therefore, despite heavy degree of coarse graining, UNRES is capable of reliable protein-structures modeling, as proved in the Community Wide Experiments on the Critical Assessment of Techniques for Protein Structure Prediction (CASP; https://www.predictioncenter.org).[15–20] Small proteins can be modeled at the *ab initio* level, while modeling larger proteins must be aided by including the information from secondary-structure and contact prediction[21] and bioinformatics model.[22,23] This need arises both from the difficulty of searching the conformational space and accumulation of errors in the structure caused by force-field inaccuracy for larger proteins. UNRES can also be run in the data-assisted mode with the information from small angle X-ray scattering (SAXS),[24] nuclear magnetic resonance (NMR)[25] or chemical cross-link mass

spectroscopy (XL-MDS)[26,27] experiments converted into the respective terms of the penalty function. Recently a version optimized for speed and memory of the UNRES package has been developed,[28] which uses the MPI and OpenMP and, subsequently, for GPUs.[29,30] Owing to these latest developments, UNRES can now run about 1 ns/day (which is equivalent to about 1 $\mu$s of laboratory time) of molecular dynamics simulations for a 232,260-residue microtubulin system. Due to the averaging of the degrees of freedom that are not considered in UNRES one time unit of UNRES is equivalent to about 1,000 time units of laboratory time.[28] UNRES is available from the dedicated web page (https://unres.pl), which also includes a link to server version[31] recently enhanced with the data-assisted-modeling capacity.[32]

Protein-structure modeling has long been the primary application of UNRES. However, the scope of applications gradually shifted to studying protein-folding and assembly pathways and kinetics,[33–41] including those involving the formation of disulfide bonds,[11,42] free-energy landscapes,[43–45] and in a variety of biology-related studies, including amyloid formation,[46–49] signaling,[50] Hsp70 chaperone cycle,[51] iron-sulfur cluster biogenesis,[52] stability of proteins from *H. pylori*,[53] and structure and dynamics of natively unfolded proteins and peptides,[54,55] the dynamics of human-norovirus-like particles[56] and long-time dynamics of molecular rotatory motors.[57] A short description of selected examples can be found in recent book chapters.[1,6]

# Derivation of the formulas for the torsional and improper-torsional potentials

## Detailed expression for the square of the distance between the atoms of different coarse-grained sites

Detailed expressions for the components of the square of the distance, $\rho^2_{Ii,Jj}$ between atom $i$ of coarse-grained site $I$ and atom $j$ of coarse-grained site $J$ (eq 3 of the main text) are given

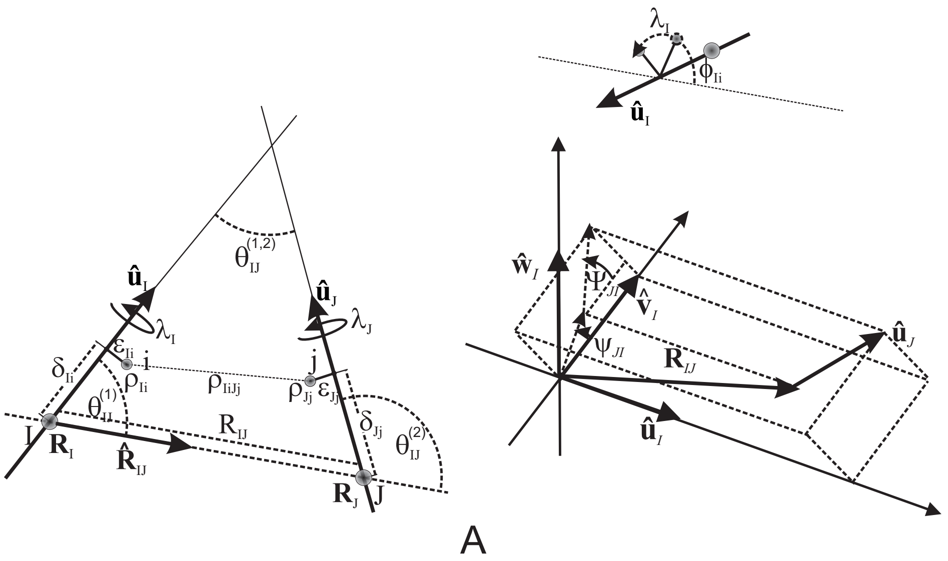


Figure S1: Illustration of the definitions of the geometric parameters of eqs S3–S13 to express the distance between atoms $i$ (with Cartesian coordinates $\boldsymbol{\rho}_{Ii}$) and $j$ (with Cartesian coordinates $\boldsymbol{\rho}_{Jj}$) of coarse-grained sites $I$ (with Cartesian coordinates $\mathbf{R}_I$) and $J$ (with the Cartesian coordinates $\mathbf{R}_J$), respectively (indicated in panel A). The centers of the of the sites are shown as larger shaded spheres and the atoms are shown as smaller shaded spheres in panels A and B. (A) Location of the sites in space. $R_{IJ}$ and $\rho_{IiJj}$ are the distances between the centers of sites $I$ and $J$ and atom $i$ of site $I$ and atom $J$ of site $J$, respectively, $\hat{\mathbf{u}}_I$ and $\hat{\mathbf{u}}_J$ are the unit vectors of the virtual-bond axes of the sites, and $\hat{\mathbf{R}}_{IJ}$ is the unit vector pointing from site $I$ to site $J$. The angles $\theta_{IJ}^{(1)}$, $\theta_{IJ}^{(2)}$, and $\theta_{IJ}^{(12)}$ are the angles between vectors $\hat{\mathbf{u}}_I$ and $\hat{\mathbf{R}}_{IJ}$, between $\hat{\mathbf{u}}_J$ and $\hat{\mathbf{R}}_{IJ}$, and between $\hat{\mathbf{u}}_I$ and $\hat{\mathbf{u}}_J$, respectively. The projection of the position of atom $i$ on the virtual-bond axis of site $I$ is $\delta_{Ii}$ and can be positive or negative depending on whether the atom is to the left or to the right of the center, and that of atom $i$ is $\delta_{Jj}$. The distance of atom $i$ from the virtual-bond axis is $\varepsilon_{Ii}$ and that of atom $j$ is $\varepsilon_{Jj}$. The angles $\lambda_I$ and $\lambda_J$ are the angles for counterclockwise rotation about the respective virtual-bond axes. (B) Illustration of the phase angle $\varphi_{Ii}$ of atom $i$ of site $I$; this angle defines the counterclockwise rotation of this atom about the virtual-bond axis for $\lambda_I = 0$. (C) Illustration of the angles $\Psi_{JI}$ and $\psi_{JI}$. $\hat{\mathbf{u}}_I$ (the unit vector of the virtual-bond axis of site $I$), $\hat{\mathbf{v}}_I$, and $\hat{\mathbf{w}}_I$ are the unit vectors of the axes of the right-handed local Cartesian-coordinate system of site $I$. $\Psi_{JI}$ and $\psi_{JI}$ are the angles of counterclockwise rotation of the projections of $\hat{\mathbf{u}}_J$ and $\hat{\mathbf{R}}_{IJ}$, respectively, onto the $\hat{\mathbf{v}}_I, \hat{\mathbf{w}}_I$ plane from the $\hat{\mathbf{v}}_I$ axis. Adapted with permission from Sieradzan et al., J. Chem. Phys., 146, 124106 (2017), Copyright 2001 AIP Publishing LLC.

by eqs 32–35 of ref 5. Because these expressions are used in the derivation of the torsional and improper-torsional potentials presented in this paper, for the sake of clarity they are recalled below (eqs S3 – S6).

$$
\begin{aligned}
d_{IiJj} &= \delta_{Ii}^2 + \delta_{Jj}^2 + \varepsilon_{Ii}^2 + \varepsilon_{Jj}^2 - 2R_{IJ}\left[\delta_{Ii}\cos\theta_{IJ}^{(1)} - \delta_{Jj}\cos\theta_{IJ}^{(2)}\right] \\
&\quad -2\delta_{Ii}\delta_{Jj}\cos\theta_{IJ}^{(12)} && \text{(S3)} \\
f_{IiJj}(\lambda_J) &= 2\varepsilon_{Jj}[R_{IJ}\sin\theta_{IJ}^{(2)}\cos(\lambda_J + \varphi_{Jj} - \psi_{IJ}) \\
&\quad -\delta_{Ii}\sin\theta_{IJ}^{(12)}\cos(\lambda_J + \varphi_{Jj} - \Psi_{IJ})] && \text{(S4)} \\
f_{JjIi}(\lambda_I) &= 2\varepsilon_{Ii}[R_{JI}\sin\theta_{JI}^{(2)}\cos(\lambda_I + \varphi_{Ii} - \psi_{JI}) - \delta_{Jj}\sin\theta_{JI}^{(12)}\cos(\lambda_I + \varphi_{Ii} - \Psi_{JI})] \\
&= -2\varepsilon_{Ii}[R_{IJ}\sin\theta_{IJ}^{(1)}\cos(\lambda_I + \varphi_{Ii} - \psi_{JI}) \\
&\quad +\delta_{Jj}\sin\theta_{IJ}^{(12)}\cos(\lambda_I + \varphi_{Ii} - \Psi_{JI})] && \text{(S5)} \\
g_{IiJj}(\lambda_I, \lambda_J) &= \varepsilon_{Ii}\varepsilon_{Jj}\left\{\left(1 - \cos\theta_{IJ}^{(12)}\right)\cos\left[(\lambda_I + \varphi_{Ii} - \Psi_{JI}) + (\lambda_J + \varphi_{Jj} - \Psi_{IJ})\right]\right. \\
&\quad \left. -\left(1 + \cos\theta_{IJ}^{(12)}\right)\cos\left[(\lambda_I + \varphi_{Ii} - \Psi_{JI}) - (\lambda_J + \varphi_{Jj} - \Psi_{IJ})\right]\right\} && \text{(S6)}
\end{aligned}
$$

with

$$
\begin{aligned}
\theta_{IJ}^{(1)} &= \arccos(\hat{\mathbf{R}}_{IJ} \circ \hat{\mathbf{u}}_I) = \pi - \theta_{JI}^{(2)} && \text{(S7)} \\
\theta_{IJ}^{(2)} &= \arccos(\hat{\mathbf{R}}_{IJ} \circ \hat{\mathbf{u}}_I) = \pi - \theta_{JI}^{(1)} && \text{(S8)} \\
\theta_{IJ}^{(12)} &= \arccos(\hat{\mathbf{u}}_I \circ \hat{\mathbf{u}}_J) = \theta_{JI}^{(12)} && \text{(S9)} \\
\Psi_{IJ} &= \operatorname{atan2}(\hat{\mathbf{w}}_J \circ \hat{\mathbf{u}}_I, \hat{\mathbf{v}}_J \circ \hat{\mathbf{u}}_I) && \text{(S10)} \\
\Psi_{JI} &= \operatorname{atan2}(\hat{\mathbf{w}}_I \circ \hat{\mathbf{u}}_{\mathbf{J}}, \hat{\mathbf{v}}_I \circ \hat{\mathbf{u}}_J) && \text{(S11)} \\
\psi_{IJ} &= \operatorname{atan2}(\hat{\mathbf{w}}_J \circ \hat{\mathbf{R}}_{IJ}, \hat{\mathbf{v}}_J \circ \hat{\mathbf{R}}_{IJ}) && \text{(S12)} \\
\psi_{JI} &= \operatorname{atan2}(\hat{\mathbf{w}}_I \circ \hat{\mathbf{R}}_{IJ}, \hat{\mathbf{v}}_I \circ \hat{\mathbf{R}}_{IJ}) && \text{(S13)}
\end{aligned}
$$

and

$$\operatorname{atan2}(y,x)=\begin{cases}\arctan\left(\frac{y}{x}\right) & \text{if } x>0\\ \arctan\left(\frac{y}{x}\right)+\pi & \text{if } x<0 \text{ and } y\geq 0\\ \arctan\left(\frac{y}{x}\right)-\pi & \text{if } x<0 \text{ and } y<0\\ \frac{\pi}{2} & \text{if } x=0 \text{ and } y>0\\ -\frac{\pi}{2} & \text{if } x=0 \text{ and } y<0\\ \text{undefined} & \text{if } x=0 \text{ and } y=0\end{cases} \tag{S14}$$

$\theta_{IJ}^{(1)}$ and $\theta_{IJ}^{(2)}$ are the angles between the virtual-bond axis of site $I$ and the direction from site $I$ to site $J$ and between the virtual-bond axis of site $J$ and the direction from site $I$ to site $J$, respectively, and $\theta_{IJ}^{(12)}$ is the angle between the virtual-bond vector of site $I$ and that of site $J$ (Figure S1A). The angle $\varphi_{Ii}$ describes the reference orientation of atom $i$ of site $I$; if the group is planar (such as, e.g., the peptide group), $\varphi_{Ii}$ is 0 or 180° (Figure S1B). The angle $\psi_{JI}$ is the angle between the projection of the $\hat{\mathbf{R}}_{IJ}$ vector on the $\hat{\mathbf{v}}_I, \hat{\mathbf{w}}_I$ plane (the yz plane of the local coordinate system of site $I$) and the vector $\hat{\mathbf{v}}_I$, while the angle $\Psi_{JI}$ is the angle of the projection of the $\hat{\mathbf{u}}_J$ vector on that plane and the vector $\hat{\mathbf{v}}_I$ (Figure S1C); the angles $\psi_{IJ}$ and $\psi_{IJ}$ are defined likewise by replacing $\hat{\mathbf{u}}_I$ with $\hat{\mathbf{u}}_J$, $\hat{\mathbf{v}}_I$ with $\hat{\mathbf{v}}_J$ and $\hat{\mathbf{w}}_I$ with $\hat{\mathbf{w}}_J$.

## Torsional and improper-torsional potentials of order 2

Although only the improper-torsional potentials are new in this paper with respect to our previous work on the scale-consistent approach to the derivation of energy expressions in the coarse-grained force fields, in order to illustrate the considerations of the main text, we also present the derivation of the regular torsional potentials.

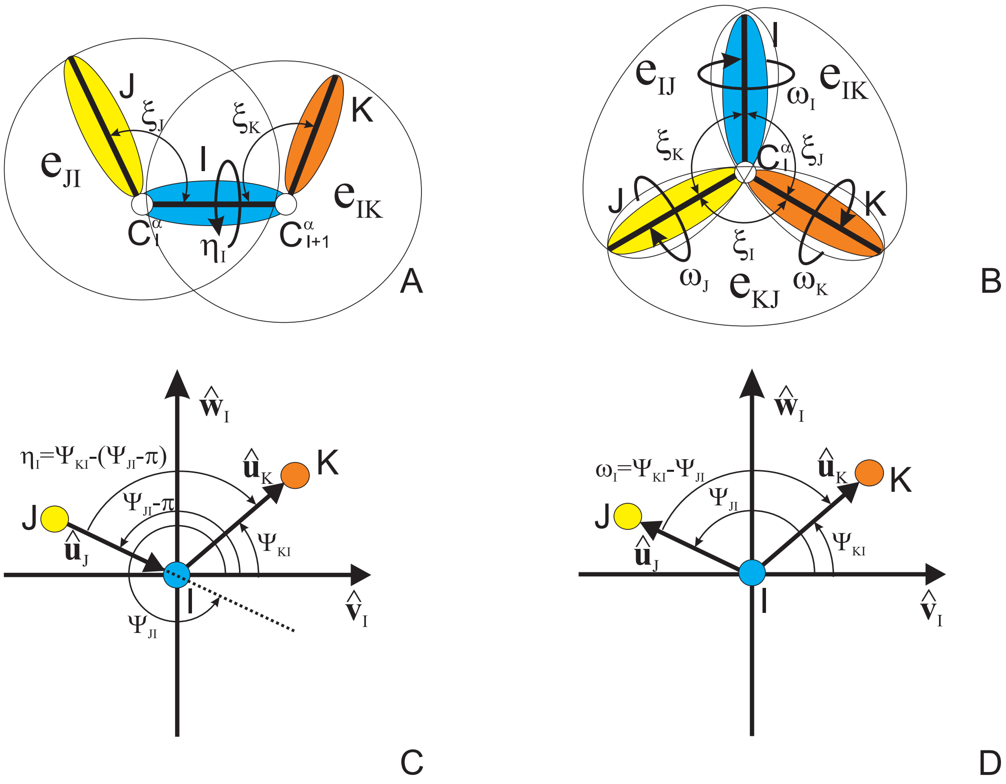


Figure S2: Illustration of the derivation of regular (A and C) and improper (B and D) torsional potentials. The torsional potentials result from the coupling of the interaction-energy surfaces of site $I$ with site $J$, $e_{IJ}$, and that of site $I$ with site $K$, $e_{IK}$. The second-order improper-torsional potentials result from the coupling between the energy surfaces of sites $I$ and $J$, sites $J$ and $K$, and sites $K$ and $I$, while the third-order improper-torsional potentials result from the coupling of the energy surfaces of all pairs of the three sites shown in panel B of the Figure. In panels C and D, the relationship between the angles $\Psi_{JI}$, which is the angle that the projection of the unit vector $\hat{\mathbf{u}}_J$ of the virtual-bond axis of site $J$ on the plane perpendicular to the virtual-bond axis of site $I$ (defined by unit vectors $\hat{\mathbf{v}}_I$ and $\hat{\mathbf{w}}_I$) and $\Psi_{KI}$, which is the angle that the projection of the unit vector $\hat{\mathbf{u}}_K$ with that plane and the regular and improper dihedral angles $\eta_I$ and $\omega_I$. The shift of $\Psi_{JI}$ in panel C by $\pi$ is due to the fact that the virtual-bond axis of site $J$ is directed towards site $I$ if the three sites are linked consecutively. Conversely, in panel D (for the improper torsional angle) the virtual-bond axis of site $J$ is directed from site $I$ (because all three sites share a common anchor point) and no phase shift has to be applied. It should be noted that the dihedral and improper-dihedral angles $\eta$ and $\omega$, respectively, are negative in panels C and D (clockwise rotation) to illustrate the subtraction of the $\Psi$ angles. The other improper-torsional angles shown in panel B can be visualised by circularly permuting the site indices in panel D.

We will index the three consecutively linked sites with the common $C^{\alpha}_{I} \cdots C^{\alpha}_{I+1}$ virtual-bond axis with $J$ (indexing the peptide group left to $C^{\alpha}_{I}$ or $SC_I$), $I$ (the peptide group between $C^{\alpha}_{I}$ and $C^{\alpha}_{I+1}$) and $K$ (the peptide group right to $C^{\alpha}_{I+1}$ or $SC_{I+1}$), as shown in

Figure S2A. For consecutively connected sites $J$, $I$, and $K$, the vectors $\mathbf{R}_{JI}$ and $\mathbf{R}_{KI}$ are in the planes defined by sites $J$ and $I$ and sites $I$ and $K$, respectively. Consequently, $\psi_{JI} = \Psi_{JI}$ and $\psi_{KI} = \Psi_{KI}$. To simplify further considerations, we locate the reference point of site $J$ in its end away from $C_I^\alpha$ (it will be $C_{I-1}^\alpha$ if site $J$ is the the peptide group preceding $C_I^\alpha$ or the respective sidechain center if it is the $I$th sidechain), that of site $I$ (which is a peptide group) in $C_I^\alpha$ and that of site $K$ in $C_{I+1}^\alpha$, respectively. With this choice, $\theta_{JI}^{(1)} = \theta_{IK}^{(1)} = 0$, $\theta_{JI}^{(2)} = \theta_{JI}^{(12)} = \pi - \xi_J$, and $\theta_{IK}^{(2)} = \theta_{IK}^{(12)} = \pi - \xi_K$ The derivation can also be carried out with setting the reference points of all sites at their centers yielding the same results but such a choice results in unnecessary complications. More discussion is presented in section IIIA of ref 5.

The cluster-cumulant expansion of the torsional PMF (eq 4 of the main text) is given by eq S15 (for clarity sake the variables are omitted).

$$W_{tor}^{JIK} = -\frac{\beta}{2}\left(\langle e_{IJ} e_{IK}\rangle - \langle e_{IJ}\rangle\langle e_{IK}\rangle\right) + \ldots = \beta U_{tor}^{JIK} \tag{S15}$$

where $\beta = 1/RT$ ($R$ being the universal gas constant and $T$ the absolute temperature)

The lowest-order cluster-cumulant contributing to the torsional term is expressed by eq S16.

$$U_{tor}^{JIK} \approx -\frac{1}{2}\sum_{ijkl} \left\langle e'_{Jj,Ii}(\rho^2_{Ji,Ij}) e'_{Ik,Kl}(\rho^2_{Ik,Kl}) \langle f_{Ji,Ij}(\lambda_I) f_{Kl,Ik}(\lambda_I)\rangle_{\lambda_I} \right\rangle_{\boldsymbol{\chi}_J,\boldsymbol{\chi}_I,\boldsymbol{\chi}_K} =$$
$$-\frac{1}{2}\sin\xi_J \sin\xi_K \times$$
$$\sum_{ijkl} \left\langle e'_{Jj,Ii}(\rho^2_{Jj,Ii}) e'_{Ik,Kl}(\rho^2_{Ik,Kl}) a_{JIK;ijkl} \langle \cos(\lambda_I + \varphi_{Ij} - \Psi_{JI}) \cos(\lambda_I + \varphi_{Ik} - \Psi_{KI})\rangle_{\lambda_I} \right\rangle_{\boldsymbol{\chi}_J,\boldsymbol{\chi}_I,\boldsymbol{\chi}_K} \tag{S16}$$

with

$$a_{JIK;ijkl} = 4\varepsilon_{Ij}\varepsilon_{Ik}(d_{JI} - \delta_{Ji})(d_{IK} - \delta_{Kl}) \tag{S17}$$

where $d_{JI}$ and $d_{IK}$ are the virtual-bond lengths of sites $J$ and $K$, respectively, and $e'(\rho^2_{Ji,Ij})$ and $e'(\rho^2_{Ik,Kl})$ are the derivatives of the interaction energy between atom $i$ of site $J$ and atom $j$ of site $I$ or between atom $k$ of site $I$ and atom $l$ of site $K$, respectively, calculated at $\rho^2_{Ji,Ij} = d^2_{JI}$ and $\rho^2_{Ik,Kl} = d^2_{IK}$, respectively. The brackets $\langle \ldots \rangle_{\lambda_I}$ and $\langle \ldots \rangle_{\boldsymbol{\chi}_J,\boldsymbol{\chi}_I,\boldsymbol{\chi}_K}$ denote the average over the collective-rotation angle $\lambda_I$ of the atoms of site $I$ about its virtual bond and that over the internal degrees of freedom of sites $J$, $I$, and $K$, respectively. Averaging over the internal degrees of freedom matters if $J$ and $K$ are flexible sidechains. For a peptide group, which has an effectively fixed geometry and for non-flexible sidechains (e.g., alanine, valine, and threonine), this averaging can be omitted or done using the harmonic approximation. Because site $I$ always is a peptide group, which is highly rigid, in what follows we omit the averaging over $\boldsymbol{\xi}_I$.

The averages of the cosine product over $\lambda_I$ are expressed by eq S18.

$$\begin{aligned}\langle \cos(\lambda_I + \varphi_{Ij} - \Psi_{JI}) \cos(\lambda_I + \varphi_{Ik} - \Psi_{KI}) \rangle_{\lambda_I} &= \frac{1}{2} \cos[(\varphi_{Ij} - \Psi_{JI}) - (\varphi_{Ik} - \Psi_{KI})] \\ &= -\frac{1}{2} \cos(\eta_I - \Delta_{I;jk}) \end{aligned} \tag{S18}$$

$$\eta_I = \Psi_{KI} - \Psi_{JI} - \pi \tag{S19}$$

$$\Delta_{I;jk} = \varphi_{Ik} - \varphi_{Ij} \tag{S20}$$

where $\eta_I$ is the virtual-bond dihedral angle defined by the virtual-bond axes of sites $J$, $I$, and $K$ and $\Delta_{I;jk}$ is the angle for the counterclockwise rotation of atom $k$ of site $I$ from atom $j$ of site $I$ about the virtual-bond axis of this site.

Finally, the lowest-order contribution to the torsional potential is expressed by eq S21

$$U_{tor}^{JIK}(\eta_I, \xi_J, \xi_K) \approx \frac{1}{4} \sin\xi_J \sin\xi_K \sum_{ijkl} e'_{Jj,Ii}(\rho^2_{Ji,Ij}) e'_{Ik,Kl}(\rho^2_{Ik,Kl}) \langle a_{JIK;ijkl} \cos(\eta_I - \Delta_{I;jk})\rangle_{\boldsymbol{\chi}_J,\boldsymbol{\chi}_K} = \frac{1}{4} A_{JIK} \sin\xi_J \sin\xi_K \cos\eta_I \tag{S21}$$

$$A_{JIK} = \sum_{ijkl} e'_{Jj,Ii}(\rho^2_{Ji,Ij}) e'_{Ik,Kl}(\rho^2_{Ik,Kl}) \langle a_{JIK;ijkl} \cos\Delta_{I;jk}\rangle_{\boldsymbol{\chi}_J,\boldsymbol{\chi}_K} \tag{S22}$$

Because site $I$ is the planar peptide group $I$ (located between $C^\alpha_I$ and $C^\alpha_{I+1}$), $\Delta_{I;jk}$ is equal either to 0 or to $\pi$ and, consequently $\cos\Delta_{I;jk}$ equals 1 or -1 and $\cos\Delta_{I;jk} = 0$. Thus, only the cosine term remains in eq S21 and the expression it symmetric. The possible averaging over the conformation of sidechain $J$ or $K$ (if any of those sites is a sidechain) involves only averaging of the projections of the positions of their atoms on the respective virtual-bond axis.

The above derivation can be extended to higher-order terms. Technically, it is better to use the second-order Fourier expansion of the the energy surfaces of systems composed of connected sites $J$ and $I$ and $I$ and $K$, respectively, as done in our earlier work.[5,12] Either way finally leads to eqs 7 – 10 of the main text. From eq S22 it is also clear that the coefficient $A_{JIK}$ can be decomposed into a contribution pertaining to the $I - J$ and that pertaining to the $J - K$ system. In our earlier work on residue-based torsional potentials we noted that the parameters can be expressed in terms of the contributions from single residue kinds.[5,12] Our present result matches the earlier one because a two linked sites can be considered a residue.

The derivation of the improper-torsional potential pertaining to the coupling between the interaction-energy surfaces of the pairs of sites attached to a common anchor point (Figure S2B) is similar. Let us consider the coupling of the interactions of site $I$ with site $J$ and those of site $I$ with site $K$. It should be noted that the improper-dihedral angle $\omega_I = \Psi_{KI} - \Psi_{JI}$ (i.e., the angle $\Psi_{JI}$ is not shifted by $\pi$). This is because all three sites are anchored to $C^\alpha_I$ and, thus, all virtual-bond axes point away from $C^\alpha_I$ (Figure S2D). Also, site $I$ can now be

a flexible side chain so the averaging might have to be carried out over its conformations.

For the improper-dihedral angles $\omega_I^{(1)}$ and $\omega_I^{(2)}$, the rotation axis is the virtual-bond of a peptide group $\mathrm{p}_{I-1}$ and $\mathrm{p}_I$, respectively (see Figure 3B of the main text). Thus, the respective second-order improper-torsional potentials are symmetric. However, for the $\omega_I^{(3)}$ angle, the rotation axis is the $\mathrm{C}_I^\alpha \cdots \mathrm{SC}_I$ axis. The sidechains are never planar and, therefore, the $\Delta_{I;jk}$ angles (eq S20) are not necessarily zero or $\pi$. However, it must be remembered that, apart from summing over all atom indices, we need to sum over all side-chain conformations to get the $U_{imp-2}(\omega^{(3)}, o_i^{(1)}, o_I^{(2)})$ improper-torsional potential.

$$
\begin{aligned}
U_{imp-2}^{p_{I-1}SC_Ip_I}(\omega_I^{(3)}, o_i^{(1)}, o_I^{(2)}) &= \frac{1}{4}\sum_{ijkl} e'_{p_{I-1}i,SC_Ij}(\rho^2_{p_{I-1}i,SC_Ij})e'_{SC_Ik,p_Il}(\rho^2_{SC_Ik,p_Il})\times \\
&\quad \langle a_{JIK;ijkl}\cos(\omega_I^{(3)} - \Delta_{SC_I;jk})\rangle_{\boldsymbol{\chi}_I} \\
&= \frac{1}{4}\sum_{ijkl} e'_{p_{I-1}i,SC_Ij}(\rho^2_{p_{I-1}i,SC_Ij})e'_{SC_Ik,p_Il}(\rho^2_{SC_Ik,p_Il})\times \\
&\quad \langle a_{JIK;ijkl}[\cos\omega_I^{(3)}\cos\Delta_{SC_I;jk} - \sin\omega_I^{(3)}\sin\Delta_{I;jk}]\rangle_{\boldsymbol{\chi}_I}
\end{aligned}
\tag{S23}
$$

If the phase shift $\Delta_{I;jk}$ results from the rotation(s) about rotatable bond(s), then the rotation(s) in opposite direction will result in the opposite phase shift and the sine terms in eq S23 will cancel out upon averaging over sidechain conformations. If $\Delta_{I;jk}$ is the phase shift of two atoms on two branches of a sidechain (e.g., the two methyl carbon atoms of a valine sidechain), the phase shift is fixed. However, in this case, we must remember that the atom pairs $Ji, Ij$, $Ik, Kl$ and $Ji, Ik$, $Ij, Kl$ must be considered in the summation of eq S23. If atoms $Ij$ and $Ik$ and their surroundings are identical (i.e., the sidechain is symmetric), the respective $\Delta$s are opposite and the sine terms cancel out. This result can be extended to more complex symmetric branched groups. Thus, the asymmetry (the sine term) of the $U_{imp-2}^{p_{I-1}SC_Ip_I}(\omega_I^{(3)}; o_i^{(1)}, o_I^{(2)})$ potential will only occur for intrinsically chiral sidechains, which are isoleucine and threonine. Nevertheless, the threonine sidechain is small and the isoleucine

sidechain is highly flexible. Therefore, assuming that the improper-torsional potentials are nearly symmetric also in these two cases seems to be a reasonable approach.

## Improper-torsional potentials of order 3

The torsional contribution to the effective energy originates from the PMF factor defined, together with its lowest-order cluster-cumulant term, by eq S24 (for clarity sake the variables are omitted).

$$
\begin{aligned}
W_{imp-3;I} = &-\frac{1}{\beta}\ln\langle\exp\left[-\beta\left(e_{p_{I-1},p_I}+e_{p_{I-1},SC_I}+e_{p_I,SC_I}\right)\right]\rangle+\frac{1}{\beta}\ln\langle\exp\left[-\beta\left(e_{p_{I-1},p_I}+e_{p_{I-1},SC_I}\right)\right]\rangle\\
&+\frac{1}{\beta}\ln\langle\exp\left[-\beta\left(e_{p_{I-1},p_I}+e_{p_I,SC_I}\right)\right]\rangle+\frac{1}{\beta}\ln\langle\exp\left[-\beta\left(e_{p_{I-1},SC_I}+e_{p_I,SC_I}\right)\right]\rangle\\
&-2\left\{\frac{1}{\beta}\ln\langle\exp\left(-\beta e_{p_{I-1},p_I}\right)\rangle+\frac{1}{\beta}\ln\langle\exp\left(-\beta e_{p_{I-1},SC_I}\right)\rangle+\frac{1}{\beta}\ln\langle\exp\left(-\beta e_{p_I,SC_I}\right)\rangle\right\}=\\
&\frac{\beta^2}{6}\left[\langle e_{p_{I-1},p_I}e_{p_{I-1},SC_I}e_{p_I,SC_I}\rangle-\langle e_{p_{I-1},p_I}e_{p_{I-1},SC_I}\rangle\langle e_{p_I,SC_I}\rangle-\langle e_{p_{I-1},p_I}e_{p_I,SC_I}\rangle\langle e_{p_{I-1},SC_I}\rangle\right.\\
&\left.-\langle e_{p_{I-1},SC_I}e_{p_I,SC_I}\rangle\langle e_{p_{I-1},p_I}\rangle+2\langle e_{p_{I-1},p_I}\rangle\langle e_{p_{I-1},SC_I}\rangle\langle e_{p_I,SC_I}\rangle\right]+\ldots=\beta^2 U_{imp-3;I}
\end{aligned}
\tag{S24}
$$

where $e_{p_{I-1},p_I}$, $e_{p_{I-1},SC_I}$, and $e_{p_I,SC_I}$ denote the interaction-energy surfaces of the peptide groups attached to $C_I^\alpha$ and those of one of those peptide groups and the sidechain attached to $C_I^\alpha$.

The combinations of averages of local-interaction energies and their products leaves only the terms with $\langle f_{Ii,Jj}g_{Jk,Kl}f_{In,Km}\rangle_{\lambda_J,\lambda_K}$ and those with $\langle g_{Ii,Jj}g_{Jk,Kl}g_{Km,In}\rangle_{\lambda_I,\lambda_J,\lambda_K}$, where $I$, $J$, and $K$ are the indices of the sites connected to $C_I^\alpha$ (see Figure S2C and D) and $i-n$ are atom indices. Here we derive only the formulas for the above-mentioned averages. Converting them to the improper-torsional-potential term, including the averaging over sidechain conformations is similar to those of second-order torsional potentials. Because only the $\lambda$ angles will be averaged out, we omit the subscript in the bracket notation.

The $\langle f_{Ii,Jj}g_{Jk,Kl}f_{In,Km}\rangle$ term is derived as given by eq S25.

$$\langle f_{Ii,Jj} g_{Jk,Kl} f_{In,Km} \rangle = -4\varepsilon_{Jj}\varepsilon_{Jk}\varepsilon_{Kl}\varepsilon_{Km}(d_I - \delta_{Ii})(d_I - \delta_{In}) \sin\xi_J \sin\xi_K$$
$$\langle \cos(\lambda_J + \varphi_{Jj} - \Psi_{IJ})\{(1-\cos\xi_I)\cos[(\lambda_J + \varphi_{Jk} - \Psi_{KJ}) + (\lambda_K + \varphi_{Kl} - \Psi_{JK})]$$
$$-(1+\cos\xi_I)\cos[(\lambda_J + \varphi_{Jk} - \Psi_{KJ}) - (\lambda_K + \varphi_{Kl} - \Psi_{JK})]\}\cos(\lambda_K + \varphi_{Km} - \Psi_{IK})\rangle =$$
$$-b_{IJK;ijklmn} \sin\xi_J \sin\xi_K \{(1-\cos\xi_I)\langle \cos(\lambda_J + \varphi_{Jj} - \Psi_{IJ}) \times$$
$$\cos[(\lambda_J + \varphi_{Jk} - \Psi_{KJ}) + (\lambda_K + \varphi_{Kl} - \Psi_{JK})]\cos(\lambda_K + \varphi_{Km} - \Psi_{IK})\rangle$$
$$-(1+\cos\xi_I)\langle \cos(\lambda_J + \varphi_{Jj} - \Psi_{IJ}) \times$$
$$\cos[(\lambda_J + \varphi_{Jk} - \Psi_{KJ}) + (\lambda_K + \varphi_{Kl} - \Psi_{JK})]\cos(\lambda_K + \varphi_{Km} - \Psi_{IK})\rangle\} =$$
$$-\frac{b_{IJK;ijklmn}}{4} \sin\xi_J \sin\xi_K \{(1-\cos\xi_I)\cos[(\Psi_{IJ} - \Psi_{KJ}) - (\varphi_{Jj} - \varphi_{Jk}) - (\Psi_{JK} - \Psi_{IK}) - (\varphi_{Kl} - \varphi_{Km})]$$
$$-(1+\cos\xi_I)\cos[(\Psi_{IJ} - \Psi_{KJ}) - (\varphi_{Jj} - \varphi_{Jk}) + (\Psi_{JK} - \Psi_{IK}) + (\varphi_{Kl} - \varphi_{Km})]\} =$$
$$\frac{b_{IJK;ijklmn}}{4} \sin\xi_J \sin\xi_K \{(1+\cos\xi_I)\cos[(\omega_J + \Delta_{J;kj}) + (\omega_K - \Delta_{K;ml})]$$
$$-(1-\cos\xi_I)\cos[(\omega_J - \Delta_{J;kj}) - (\omega_K - \Delta_{K;ml})]\} \quad \text{(S25)}$$

with

$$b_{IJK;ijklmn} = 4\varepsilon_{Jj}\varepsilon_{Jk}\varepsilon_{Kl}\varepsilon_{In}(d_I - \delta_{Ii})(d_I - \delta_{In}) \quad \text{(S26)}$$

and we note that (see Figure S2D)

$$\Psi_{KI} - \Psi_{JI} = \omega_I \quad \text{(S27)}$$
$$\Psi_{IJ} - \Psi_{KJ} = \omega_J \quad \text{(S28)}$$
$$\Psi_{JK} - \Psi_{IK} = \omega_K \quad \text{(S29)}$$

For the sites attached to $C_I^\alpha$, there are three terms of the form given by eq S25, which can be obtained by setting $I = p_{I-1}$, $J = p_I$, $K = SC_I$, then $I = p_I$, $J = SC_I$, $K = p_{I-1}$

and, finally, $I = SC_I$, $J = p_{I-1}$, $K = p_I$, respectively and renaming the $\xi_I$, $\xi_J$ and $\xi_K$ planar angles and the $\omega_I$, $\omega_J$, and $\omega_K$ improper-dihedral angles after the definitions of Figure 3B of the main text. Because the phase angles $\Delta$ are 0 or $\pi$ in most cases (except for the $\omega^{(3)}$ angles of isoleucine and threonine), we obtain the terms of the first part of eq 14 of the main text.

The expression for $\langle g_{Ii,Jj} g_{Jk,Kl} g_{Km,In} \rangle$ is derived as shown below.

$$\langle g_{Ii,Jj} g_{Jk,Kl} g_{Km,In} \rangle = -\varepsilon_{Ii}\varepsilon_{Jj}\varepsilon_{Jk}\varepsilon_{Kl}\varepsilon_{Km}\varepsilon_{In}$$

$$\langle\{(1-\cos\xi_I)\cos[(\lambda_I+\varphi_{Ii}-\Psi_{JI})+(\lambda_J+\varphi_{Jj}-\Psi_{IJ})]$$

$$-(1+\cos\xi_I)\cos[(\lambda_I+\varphi_{Ii}-\Psi_{JI})-(\lambda_J+\varphi_{Jj}-\Psi_{IJ})]\}\times$$

$$\{(1-\cos\xi_J)\cos[(\lambda_J+\varphi_{Jk}-\Psi_{KJ})+(\lambda_K+\varphi_{Kl}-\Psi_{JK})]$$

$$-(1+\cos\xi_J)\cos[(\lambda_J+\varphi_{Jk}-\Psi_{KJ})-(\lambda_K+\varphi_{Kl}-\Psi_{JK})]\}\times$$

$$\{(1-\cos\xi_K)\cos[(\lambda_K+\varphi_{Km}-\Psi_{IK})+(\lambda_I+\varphi_{In}-\Psi_{KI})]$$

$$-(1+\cos\xi_K)\cos\left[(\lambda_K+\varphi_{Km}-\Psi_{IK})-(\lambda_K+\varphi_{Km}-\Psi_{KI})\right]\}\rangle =$$

$$-c_{IJK;ijklmn}\sum_{s_I=\pm1,s_J=\pm1,s_K=\pm1} s_I s_J s_K (1-s_I\cos\xi_I)(1-s_J\cos\xi_J)(1-s_K\cos\xi_K)$$

$$\langle\cos[(\lambda_I+\varphi_{Ii}-\Psi_{JI})+s_I(\lambda_J+\varphi_{Jj}-\Psi_{IJ})]\times$$

$$\cos[(\lambda_J+\varphi_{Jk}-\Psi_{KJ})+s_J(\lambda_K+\varphi_{Kl}-\Psi_{JK})]\times$$

$$\cos[(\lambda_K+\varphi_{Km}-\Psi_{IK})+s_K(\lambda_I+\varphi_{In}-\Psi_{KI})]\rangle =$$

$$\frac{c_{IJK;ijklmn}}{4}\sum_{\substack{s_I=\pm1,s_J=\pm1,s_K=\pm1\\ s_I s_J s_K=-1}} (1-s_I\cos\xi_I)(1-s_J\cos\xi_J)(1-s_K\cos\xi_K)\times$$

$$\cos[-s_K(\varphi_{Ii}-\Psi_{JI})+s_J(\varphi_{Jj}-\Psi_{IJ})-s_J(\varphi_{Jk}-\Psi_{KJ})-$$

$$(\varphi_{Kl}-\Psi_{JK})+(\varphi_{Kn}-\Psi_{IK})+s_K(\varphi_{In}-\Psi_{KI})] =$$

$$\frac{c_{IJK;ijklmn}}{4}\sum_{\substack{s_I=\pm1,s_J=\pm1,s_K=\pm1\\ s_I s_J s_K=-1}} (1-s_I\cos\xi_I)(1-s_J\cos\xi_J)(1-s_K\cos\xi_K)\times$$

$$\cos[s_J(\omega_I - \Delta_{I;in}) + s_K(\omega_J - \Delta_{J;jk}) + s_I(\omega_K - \Delta_{K;lm})] =$$
$$\frac{c_{IJK;ijklmn}}{4}\{(1+\cos\xi_I)(1+\cos\xi_J)(1+\cos\xi_K)\cos[(\omega_I - \Delta_{I;in}) + (\omega_J - \Delta_{J;jk}) + (\omega_K - \Delta_{K;lm})]$$
$$+(1-\cos\xi_I)(1+\cos\xi_J)(1+\cos\xi_K)\cos[(\omega_I - \Delta_{I;in}) + (\omega_J - \Delta_{J;jk}) - (\omega_K - \Delta_{K;lm})]$$
$$+(1+\cos\xi_I)(1-\cos\xi_J)(1+\cos\xi_K)\cos[-(\omega_I - \Delta_{I;in}) + (\omega_J - \Delta_{J;jk}) + (\omega_K - \Delta_{K;lm})]$$
$$+(1+\cos\xi_I)(1+\cos\xi_J)(1-\cos\xi_K)\cos[(\omega_I - \Delta_{I;in}) - (\omega_J - \Delta_{J;jk}) + (\omega_K - \Delta_{K;lm})]\} \tag{S30}$$

with

$$c_{IJK;ijklmn} = \varepsilon_{Ii}\varepsilon_{Jj}\varepsilon_{Jk}\varepsilon_{Kl}\varepsilon_{Km}\varepsilon_{In} \tag{S31}$$

In the subsequent transformations of eq S30 we took advantage of the fact that the constraint $s_I s_J s_K = -1$ implies $s_I s_J = -s_K$, $s_I s_K = -s_J$ and $s_J s_K = -s_I$. By setting $J = p_{I-1}$, $K = p_I$, and $I = SC_I$ and, thereby, $\xi_I = \theta_I$, $\xi_J = o_I^{(1)}$, $\xi_K = o_I^{(2)}$, $\omega_J = \omega_I^{(1)}$, $\omega_K = \omega_I^{(2)}$, and $\omega_I = \omega_I^{(3)}$ (see Figure 3B of the main text), summing over the atomic indices, averaging over sidechain conformations, we obtain eq 14 of the main text.

# Calculation of the torsional and improper-torsional PMF surfaces

The model systems used to calculate the torsional and second-order improper-torsional potentials discussed in this work are shown in Figure S3.

For each of the three systems shown in Figure S3, the calculations were carried out with by using the PM7 semiempirical method of molecular quantum mechanics[59] with the PCM mean-field solvation model.[60] It should be noted that too short distances between the hydrogen and the oxygen atoms not involved in hydrogen bonds have recently been reported when minimizing the experimental structures of proteins with the PM7 Hamiltonian.[61] A

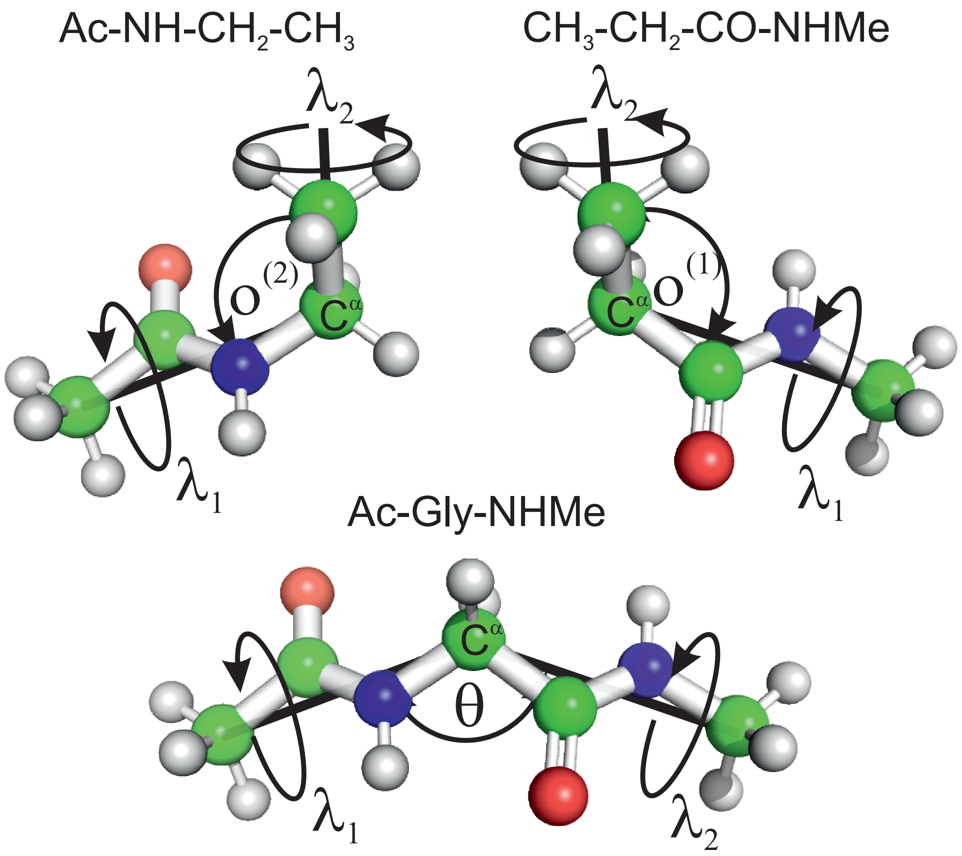


Figure S3: The Ac-Gly-NHMe (bottom) Ac-NH-$CH_2$-Me (top left) and Me-$CH_2$-CO-NHMe (top right) systems used to determine the torsional and second-order improper-torsional PMFs for polyalanine chains. The primary grid variables were the angles $\lambda_1$ and $\lambda_2$ shown in the panels but scan was also carried out in the $o^{(1)}$, $o^{(2)}$ and $\theta$ angles. It should be noted that the angles $\lambda_2$ in the upper panels govern the rotation of the rear $\alpha$-hydrogen atom ($H^{\alpha}$ of alanine) given the fixed positions of the other atoms attached to the $C^{\alpha}$ atom and not the rotation of the methyl group, to describe the distortion from tetrahedral geometry. The drawings were made with PyMol.[58]

correction to PM7 has been proposed to remove this problem.[61] However, we did not observe low-energy structures of the model compounds considered in this study when constructing the respective energy maps, which means that the problem appears only for larger systems in which crowding is significant. Moreover, it has been shown that the free energies of protein-ligand interactions are better reproduced with PM7 than with *ab initio* and DFT methods.[62] Therefore, the PM7 method seems to be sufficiently accurate for the purpose of this work.

Calculations were carried out by using the Gaussian[63] and MOPAC[64] software. A grid in $\lambda_1$ and $\lambda_2$ torsional angles ranging from $-180°$ to $165°$ and with the separation of $15°$ (576 points) was constructed and energy was minimized for each grid point with constraining the $\lambda_1$ and $\lambda_2$ angles to the respective grid values. After minimization, a scan in the respective planar angle ($\theta$, $o^{(1)}$ or $o^{(2)}$) was carried out about the respective equilibrium value in the single-point energy calculation mode with the spacing of $5°$. The torsional and improper-torsional PMFs were calculated from the potential-energy maps by numerical integration as described in our previous work.[5,12,65,66] The two-dimensional PMFs shown in Figure 4 of the main text were calculated on a grid with dihedral-angle spacing of $15°$ and planar-angle spacing of $5°$. The one-dimensional torsional potentials shown in Figure 5 of the main text were calculated by including the planar angles in the integration to calculate the PMF.

The PMF of the torsional potential resulting from the coupling of the energy surfaces of two terminally-blocked alanine residue (shown as grey circles and grey dashed line in Figure 5A of the main text) was determined as described in our earlier work[65,66] except that the PM7 method with the PCM solvation model was used to calculate the potential-energy surface of the Ac-Ala-NHMe system.

The plot of the second-order improper-torsion PMF shown in Figure 6B of the main text was obtained by summing all three second-order improper-torsional potentials, as given by eq S32 subject to the constraints resulting from the relationship between the virtual-bond angles and improper dihedral angles sharing a common vertex atom, as given by eqs S33 – S35. We expressed this total improper-torsional PMF as a function of the $\omega^{(3)}$ improper-

dihedral angle for rotation about the $C^\alpha$-$C^\beta$ bond (for alanine the direction of the $C^\alpha \cdots SC$ virtual-bond axis is that of the $C^\alpha$-$C^\beta$ bond) (eq S32) but all three improper dihedral angles were similar due to geometric constraints given by eqs S33 – S35.

$$W^{(2)}_{imp-tor}(\omega^{(3)}) = W^{(2)}_{imp-tor}(\omega^{(1)}, o^{(2)}, \theta) + W^{(2)}_{imp-tor}(\omega^{(2)}, o^{(1)}, \theta) + W^{(2)}_{imp-tor}(\omega^{(3)}, o^{(1)}, o^{(2)}) \quad \text{(S32)}$$

$$\cos\omega^{(1)} = \frac{\cos o^{(1)} - \cos o^{(2)} \cos\theta}{\sin o^{(2)} \sin\theta} \quad \text{(S33)}$$

$$\cos\omega^{(2)} = \frac{\cos o^{(2)} - \cos o^{(1)} \cos\theta}{\sin o^{(1)} \sin\theta} \quad \text{(S34)}$$

$$\cos\omega^{(3)} = \frac{\cos\theta - \cos o^{(1)} \cos o^{(2)}}{\sin o^{(1)} \sin o^{(2)}} \quad \text{(S35)}$$

# Calculation of the PMF profile of serine residue enantiomerization

To calculate the enantiomerization PMF profile, we studied the enolization pathway of a terminally-blocked L-serine residue mediated by two water molecules, as in ref 67. We assumed that the pathway from the enol form of serine to D-serine is the reversal of the pathway from the L-serine residue to its enol (achiral) form. We used the PM7 semiempirical method[59] with the PCM solvation model.[60] Calculations were carried out with the MOPAC[64] software. The MOPAC SADDLE, NLLSQ, and TS functions were used to locate and optimize the geometries of the transition states and the FORCE function was used to check the signature of a transition state. After the transition states were located, the structures on the reaction pathways were determined by calculated the Intrinsic Reaction Coordinate (the IRC function of MOPAC). The PMF profile was calculated, as a function of the $\omega^{(3)}$ improper-dihedral angle (Figure 3B in the main text) from the histogram in $\omega^{(3)}$, with a 5° bin size, collected from all IRC points and mirror-imaged about $\omega^{(3)} = 180°$ for the

part of the profile corresponding to the transition from the enol form of serine to D-serine.

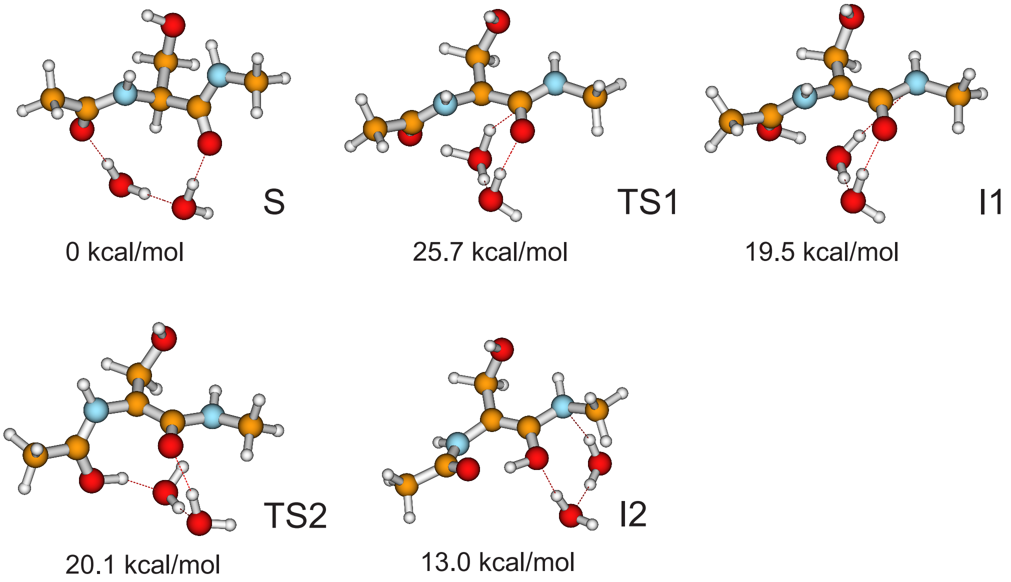


Figure S4: Half of the L,D-isomerization reaction path of L-serine mediated by two water molecule (S) leading to the enol intermediate (I2) through the zwitterionic enol form (I1) calculated with the PM7 semiempirical method[59] with the PCM water model.[60] TS1 and TS2 denote the respective transition states. The drawings were made with MOLDEN.[68]

The transition states and intermediates on the enolization pathway, along with their relative energies (with respect to L-serine plus 2 water molecules) are shown in Figure S4. As can be seen from the Figure, the reaction pathway is quite complex and proceeds through a zwitterionic intermediate (I1), in which the proton is attached to the acetyl carbonyl carbon, this resulting in a significant net positive charge on the acetyl carbon atom and a significant negative charge on the $C^{\alpha}$ atom. Upon a proton transfer, this intermediate transforms into the regular enol intermediate (I2). Attempts to fine a direct reaction path from L-serine (S) to I2 resulted in finding transition states with order higher than 1 (with more than 1 negative eigenvalue of energy Hessian).

# References


(1) Sieradzan, A. K.; Czaplewski, C.; Krupa, P.; Mozolewska, M. A.; Karczyńska, A. S.; Lipska, A. G.; Lubecka, E. A.; Gołaś, E.; Wirecki, T.; Makowski, M.; Ołdziej, S.; Liwo, A. In *Protein Folding: Methods and Protocols*; Muñoz, V., Ed.; Springer US: New York, NY, 2022; pp 399–416.

(2) Zięba, K.; Ślusarz, M.; Ślusarz, R.; Liwo, A.; Czaplewski, C.; Sieradzan, A. K. Extension of the UNRES Coarse-Grained Force Field to Membrane Proteins in the Lipid Bilayer. *J. Phys. Chem. B* **2019**, *123*, 7829–7839.

(3) Liwo, A.; Ołdziej, S.; Pincus, M. R.; Wawak, R. J.; Rackovsky, S.; Scheraga, H. A. A United-Residue Force Field for Off-Lattice Protein-Structure Simulations. I. Functional Forms and Parameters of Long-Range Side-Chain Interaction Potentials from Protein Crystal Data. *J. Comput. Chem.* **1997**, *18*, 849–873.

(4) Makowski, M.; Liwo, A.; Scheraga, H. A. Simple Physics-Based Analytical Formulas for the Potentials of Mean Force of the Interaction of Amino Acid Side Chains in Water. VII. Charged-Hydrophobic/Polar and Polar-Hydrophobic/Polar Side Chains. *J. Phys. Chem. B* **2017**, *121*, 379–390.

(5) Sieradzan, A. K.; Makowski, M.; Augustynowicz, A.; Liwo, A. A General Method for the Derivation of the Functional Forms of the Effective Energy Terms in Coarse-Grained Energy Functions of Polymers. I. Backbone Potentials of Coarse-Grained Polypeptide Chains. *J. Chem. Phys.* **2017**, *146*, 124106.

(6) Liwo, A.; Czaplewski, C.; Sieradzan, A. K.; Lubecka, E. A.; Lipska, A. G.; Golon, Ł.; Karczyńska, A.; Krupa, P.; Mozolewska, M. A.; Makowski, M.; Ganzynkowicz, R.; Giełdoń, A.; Maciejczyk, M. In *Progress in molecular biology and translational science. Computational Approaches for Understanding Dynamical Systems: Protein Folding and*

*Assembly*; Strodel, B., Barz, B., Eds.; Academic Press, London, 2020; Vol. 170; Chapter 2, pp 73–122.

(7) Kozłowska, U.; Maisuradze, G. G.; Liwo, A.; Scheraga, H. A. Determination of Side-Chain-Rotamer and Side-Chain and Backbone Virtual-Bond-Stretching Potentials of Mean Force From AM1 Energy Surfaces of Terminally-Blocked Amino-Acid Residues, for Coarse-Grained Simulations of Protein Structure and Folding. 2. Results, Comparison with Statistical Potentials, and Implementation in the UNRES Force Field. *J. Comput. Chem.* **2010**, *31*, 1154–1167.

(8) Liwo, A.; Czaplewski, C.; Pillardy, J.; Scheraga, H. A. Cumulant-Based Expressions for the Multibody Terms for the Correlation between Local and Electrostatic Interactions in the United-Residue Force Field. *J. Chem. Phys.* **2001**, *115*, 2323–2347.

(9) Kolinski, A.; Skolnick, J. Discretized Model of Proteins. I. Monte Carlo Study of Cooperativity in Homopolypeptides. *J. Chem. Phys.* **1992**, *97*, 9412–9426.

(10) Chinchio, M.; Czaplewski, C.; Liwo, A.; Ołdziej, S.; Scheraga, H. A. Dynamic Formation and Breaking of Disulfide Bonds in Molecular Dynamics Simulations with the UNRES Force Field. *J. Chem. Theory Comput.* **2007**, *3*, 1236–1248.

(11) Krupa, P.; Sieradzan, A. K.; Mozolewska, M. A.; Li, H.; Liwo, A.; Scheraga, H. A. Dynamics of Disulfide-Bond Disruption and Formation in the Thermal Unfolding of Ribonuclease A. *J. Chem. Theory Comput.* **2017**, *13*, 5721–5730.

(12) Liwo, A.; Sieradzan, A. K.; Lipska, A. G.; Czaplewski, C.; Joung, I.; Żmudzińska, W.; Hałabis, A.; Ołdziej, S. A General Method for the Derivation of the Functional Forms of the Effective Energy Terms in Coarse-Grained Energy Functions of Polymers. III. determination of Scale-Consistent Backbone-Local and Correlation Potentials in the UNRES Force Field and Force-Field Calibration and Validation. *J. Chem. Phys.* **2019**, *150*, 155104.

(13) Liwo, A.; Khalili, M.; Czaplewski, C.; Kalinowski, S.; Ołdziej, S.; Wachucik, K.; Scheraga, H. A. Modification and Optimization of the United-Residue (UNRES) Potential Energy Function for Canonical Simulations. I. Temperature Dependence of the Effective Energy Function and Tests of the Optimization Method with Single Training Proteins. *J. Phys. Chem. B* **2007**, *111*, 260–285.

(14) Borges-Araújo, L.; Patmanidis, I.; Singh, A. P.; Santos, L. H. S.; Sieradzan, A. K.; Vanni, S.; Czaplewski, C.; Pantano, S.; Shinoda, W.; Monticelli, L.; Liwo, A.; Marrink, S. J.; Souza, P. C. T. Pragmatic Coarse-Graining of Proteins: Models and Applications. *J. Chem. Theory Comput.* **2023**, *ASAP article*, DOI:10.1021/acs.jctc.3c00733.

(15) Liwo, A.; Lee, J.; Ripoll, D. R.; Pillardy, J.; Scheraga, H. A. Protein Structure Prediction by Global Optimization of a Potential Energy Function. *Proc. Natl. Acad. Sci., U. S. A.* **1999**, *96*, 5482–5485.

(16) Ołdziej, S. et al. Physics-Based Protein-Structure Prediction Using a Hierarchical Protocol Based on the UNRES Force Field: Assessment in Two Blind Tests. *Proc. Natl. Acad. Sci. U.S.A.* **2005**, *102*, 7547–7552.

(17) He, Y.; Mozolewska, M. A.; Krupa, P.; Sieradzan, A. K.; Wirecki, T. K.; Liwo, A.; Kachlishvili, K.; Rackovsky, S.; Jagieła, D.; Ślusarz, R.; Czaplewski, C. R.; Ołdziej, S.; Scheraga, H. A. Lessons from Application of the UNRES Force Field to Predictions of Structures of CASP10 Targets. *Proceedings of the National Academy of Sciences* **2013**, *110*, 14936–14941.

(18) Krupa, P. et al. Performance of Protein-Structure Predictions with the Physics-Based UNRES Force Field in CASP11. *Bioinformatics* **2016**, *32*, 3270–3278.

(19) Lubecka, E. A. et al. Evaluation of the Scale-Consistent UNRES Force Field in Template-Free Prediction of Protein Structures in the CASP13 Experiment. *J. Mol. Graph. Model.* **2019**, *92*, 154–166.

(20) Antoniak, A. et al. Modeling Protein Structures with the Coarse-Grained UNRES Force Field in the CASP14 Experiment. *J. Mol. Graph. Model.* **2021**, *108*, 108008.

(21) Lubecka, E. A.; Liwo, A. Introduction of a Bounded Penalty Function in Contact-Assisted Simulations of Protein Structures to Omit False Restraints. *J. Comput. Chem.* **2019**, *40*, 2164–2178.

(22) Mozolewska, M.; Krupa, P.; Zaborowski, B.; Liwo, A.; Lee, J.; Joo, K.; Czaplewski, C. Use of Restraints from Consensus Fragments of Multiple Server Models To Enhance Protein-Structure Prediction Capability of the UNRES Force Field. *J. Chem. Inf. Model.* **2016**, *56*, 2263–2279.

(23) Karczyńska, A.; Mozolewska, M. A.; Krupa, P.; Giełdoń, A.; Bojarski, K. K.; Zaborowski, B.; Liwo, A.; Ślusarz, R.; Ślusarz, M.; Lee, J.; Joo, K.; Czaplewski, C. Use of the UNRES Force Field in Template-Based Prediction of Protein Structures and the Refinement of Server Models: Test with CASP12 Targets. *J. Mol. Graph. Model.* **2018**, *83*, 92–99.

(24) Karczyńska, A. S.; Mozolewska, M. A.; Krupa, P.; Giełdoń, A.; Liwo, A.; Czaplewski, C. Prediction of Protein Structure with the Coarse-Grained UNRES Force Field Assisted by Small X-Ray Scattering Data and Knowledge-Based Information. *Proteins* **2018**, *86*, 228–239.

(25) Lubecka, E.; Liwo, A. A Coarse-Grained Approach to NMR-Data-Assisted Modeling of Protein Structures. *J. Comput. Chem.* **2022**, *43*, 2047–2059.

(26) Fajardo, J. E. et al. Assessment of Chemical-Crosslink-Assisted Protein Structure Modeling in CASP13. *Proteins* **2019**, *87*, 1283–1297.

(27) Kogut, M.; Gong, Z.; Tang, C.; Liwo, A. Pseudopotentials for Coarse-Grained Cross-Link-Assisted Modeling of Protein Structures. *J. Comput. Chem.* **2021**, *42*, 2054–2067.

(28) Sieradzan, A. K.; Sans-Duñó, J.; Lubecka, E. A.; Czaplewski, C.; Lipska, A. G.; Leszczynski, H.; Ocetkiewicz, K. M.; Proficz, J.; Czarnul, P.; Krawczyk, H.; Liwo, A. Optimization of Parallel Implementation of UNRES Package for Coarse-Grained Simulations to Treat Large Proteins. *J. Comput. Chem.* **2023**, *44*, 602–625.

(29) Ocetkiewicz, K. M.; Czaplewski, C.; Krawczyk, H.; Lipska, A. G.; Liwo, A.; Proficz, J.; Sieradzan, A. K.; Czarnul, P. UNRES-GPU for Physics-Based Coarse-Grained Simulations of Protein Systems at Biological Time- and Size-Scales. *Bioinformatics* **2023**, *39*, btad391.

(30) Ocetkiewicz, K. M.; Czaplewski, C.; Krawczyk, H.; Lipska, A. G.; Liwo, A.; Proficz, J.; Sieradzan, A. K.; Czarnul, P. Multi-GPU UNRES for Scalable Coarse-Grained Simulations of Very Large Protein Systems. *Comput. Phys. Commun.* **2023**, submitted.

(31) Czaplewski, C.; Karczyńska, A.; Sieradzan, A. K.; Liwo, A. UNRES Server for Physics-Based Coarse-Grained Simulations and Prediction of Protein Structure, Dynamics and Thermodynamics. *Nucl. Acids Res.* **2018**, *46*, W304–W309.

(32) Ślusarz, R.; Lubecka, E.; Czaplewski, C.; Liwo, A. Improvements and New Functionalities of UNRES Server for Coarse-Grained Modeling of Protein Structure, Dynamics, and Interactions. *Front. Biomol. Sci* **2022**, *9*, 1071428.

(33) Liwo, A.; Khalili, M.; Scheraga, H. A. Ab Initio Simulations of Protein-Folding Pathways by Molecular Dynamics with the United-Residue Model of Polypeptide Chains. *Proc. Natl. Acad. Sci. U.S.A.* **2005**, *102*, 2362–2367.

(34) Khalili, M.; Liwo, A.; Scheraga, H. A. Kinetic Studies of Folding of the B-Domain of Staphylococcal Protein A with Molecular Dynamics and a United-Residue (UNRES) Model of Polypeptide Chains. *J. Mol. Biol.* **2006**, *355*, 536–547.

(35) He, Y.; Chen, C.; Xiao, Y. United-Residue (UNRES) Langevin Dynamics Simulations of Trpzip2 Folding. *J. Comput. Biol.* **2009**, *16*, 1719–1730.

(36) Tuszynska, I.; Bujnicki, J. Predicting Atomic Details of the Unfolding Pathway for YibK, a Knotted Protein from the SPOUT Superfamily. *J. Biomol. Struct. Dyn.* **2010**, *27*, 511–520.

(37) Sieradzan, A. K.; Liwo, A.; Hansmann, U. H. E. Folding and Self-Assembly of a Small Protein Complex. *J. Chem. Theory Comput.* **2012**, *8*, 3416–3422.

(38) Gao, K.; Yang, M. Molecular Dynamics Simulations of Helix Bundle Proteins Using UNRES Force Field and All-Atom Force Field. *J. Theor. Comput. Chem.* **2012**, *11*, 1201–1215.

(39) Yin, Y.; Maisuradze, G. G.; A, L.; Scheraga, H. A. Hidden Protein Folding Pathways in Free-Energy Landscapes Uncovered by Network Analysis. *J. Chem. Theory Comput.* **2012**, *8*, 1176–1189.

(40) Zhou, R.; Maisuradze, G. G.; Suñol, D.; Todorovski, T.; Macias, M. J.; Xiao, Y.; Scheraga, H. A.; Czaplewski, C.; Liwo, A. Folding Kinetics of WW Domains with the United Residue Force Field for Bridging Microscopic Motions and Experimental Measurements. *Proc. Natl. Acad. Sci., U.S.A.* **2014**, *111*, 18243–18248.

(41) Lipska, A. G.; Seidman, S. R.; Sieradzan, A. K.; Giełdoń, A.; Liwo, A.; Scheraga, H. A. Molecular Dynamics of Protein A and a WW Domain with a United-Residue Model Including Hydrodynamic Interaction. *J. Chem. Phys.* **2016**, *144*, 184110.

(42) Smardz, P.; Sieradzan, A. K.; Krupa, P. Mechanical Stability of Ribonuclease A Heavily Depends on the Redox Environment. *J. Phys. Chem. B* **2022**, *126*, 6240âĂŞ6249.

(43) Maisuradze, G. G.; Liwo, A.; Scheraga, H. A. Principal Component Analysis for Protein Folding Dynamics. *J. Mol. Biol.* **2009**, *385*, 312–329.

(44) Maisuradze, G. G.; Senet, P.; Czaplewski, C.; Liwo, A.; Scheraga, H. A. Investigation of

Protein Folding by Coarse-Grained Molecular Dynamics with the UNRES Force Field. *J. Phys. Chem. A* **2010**, *114*, 4471–4485.

(45) Maisuradze, G. G.; Zhou, R.; Liwo, A.; Xiao, Y.; Scheraga, H. A. Effects of Mutation, Truncation, and Temperature on the Folding Kinetics of a WW Domain. *J. Mol. Biol.* **2012**, *420*, 350–365.

(46) Rojas, A.; Liwo, A.; Browne, D.; Scheraga, H. A. Mechanism of Fiber Assembly; Treatment of A$\beta$-Peptide Peptide Aggregation with a Coarse-Grained United-Residue Force Field. *J. Mol. Biol.* **2010**, *404*, 537–552.

(47) Rojas, A.; Liwo, A.; Scheraga, H. A. A Study of the $\alpha$-helical Intermediate Preceding the Aggregation of the Amino-Terminal Fragment of the A$\beta$-Amyloid Peptide (1-28). *J. Phys. Chem. B* **2011**, *115*, 12978–12983.

(48) Rojas, A.; Maisuradze, N.; Kachlishvili, K.; Scheraga, H. A.; Maisuradze, G. G. Elucidating Important Sites and the Mechanism for Amyloid Fibril Formation by Coarse-Grained Molecular Dynamics. *ACS Chem. Neurosci.* **2017**, *8*, 201–209.

(49) Rojas, A.; Maisuradze, G.; Scheraga, H. Dependence of the Formation of Tau and A Beta Peptide Mixed Aggregates on the Secondary Structure of the N-Terminal Region of A Beta. *J. Phys. Chem. B* **2018**, *122*, 7049–7056.

(50) Y. He, A. L.; Weinstein, H.; Scheraga, H. A. PDZ Binding to the BAR Domain of PICK1 is Elucidated by Coarse-Grained Molecular Dynamics. *J. Mol. Biol.* **2011**, *405*, 298–314.

(51) Golas, E. I.; Maisuradze, G. G.; Senet, P.; Ołdziej, S.; Czaplewski, C.; Scheraga, H. A.; Liwo, A. Simulation of the Opening and Closing of Hsp70 Chaperones by Coarse-Grained Molecular Dynamics. *J. Chem. Theory Comput.* **2012**, *8*, 1334–1343.

(52) Mozolewska, M. A.; Krupa, P.; Scheraga, H. A.; Liwo, A. Molecular Modeling of the Binding Modes of the Iron-Sulfur Protein to the Jac1 Co-Chaperone from Saccharomyces cerevisiae by All-Atom and Coarse-Grained Approaches. *Proteins: Structure, Function, and Bioinformatics* **2015**, *83*, 1414–1426.

(53) Grzeszczuk, M. J.; Bąk, A.; Banaś, A. M.; Urbanowicz, P.; Dunin-Horkawicz, S.; Giełdoń, A.; Czaplewski, C.; Liwo, A.; Jagusztyn-Krynicka, E. K. Impact of Selected Amino Acids of HP0377 (Helicobacter pylori Thiol Oxidoreductase) on Its Functioning as a CcmG (Cytochrome C Maturation) Protein and Dsb (Disulfide Bond) Isomerase. *PLoS ONE* **2018**, *13*, e0195358.

(54) S. D, V.; Miele, A. E.; Uciechowska-Kaczmarzyk, U.; Liwo, A.; Duclos, B.; Samsonov, S. A.; Ricard-Blum, S. Insights into the Structure and Dynamics of Lysyl Oxidase Propeptide, a Flexible Protein with Numerous Partners. *Sci. Rep.* **2018**, *8*, 11768.

(55) Biskupek, I.; Czaplewski, C.; Sawicka, J.; Iłowska, E.; Dzierżyńska, M.; Rodziewicz-Motowidło, S.; Liwo, A. Prediction of Aggregation of Biologically-Active Peptides with the UNRES Coarse-Grained Model. *Biomolecules* **2022**, *12*, 1140.

(56) Lipska, A. G.; Sieradzan, A. K.; Czaplewski, C.; Lipińska, A. D.; Ocetkiewicz, K. M.; Proficz, J.; Czarnul, P.; Krawczyk, H.; Liwo, A. Long-Time Scale Simulations of Virus-Like Particles From Three Human-Norovirus Strains. *J. Comput. Chem.* **2023**, *44*, 1470–1483.

(57) Liwo, A.; Pyrka, M.; Czaplewski, C.; Peng, X.; Niemi, A. J. Long-Time Dynamics of Selected Molecular-Motor Components Using a Physics-Based Coarse-Grained Approach. *Biomolecules* **2023**, *13*, 941.

(58) Schrödinger, LLC, The PyMOL molecular graphics system. 2010.

(59) Stewart, J. J. P. Optimization of Parameters for Semiempirical Methods VI: More

Modifications to the NDDO Approximations and Re-Optimization of Parameters. *J. Mol. Model.* **2013**, *19*, 1–32.

(60) Mennucci, B.; Tomasi, J.; Cammi, R.; Cheeseman, J. R.; Frisch, M. J.; Devlin, F. J.; Gabriel, S.; ; Stephens, P. J. Polarizable Continuum Model (PCM) Calculations of Solvent Effects on Optical Rotations of Chiral Molecules. *J. Phys. Chem. A* **2002**, *106*, 6102–6113.

(61) Řezáč, J.; Stewart, J. J. P. How Well Do Semiempirical QM Methods Describe the Structure of Proteins? *J. Chem. Phys.* **2023**, *158*, 044118.

(62) Kříž, K.; Řezáč, J. Benchmarking of Semiempirical Quantum-Mechanical Methods on Systems Relevant to Computer-Aided Drug Design. *J. Chem. Inf. Model.* **2020**, *60*, 1453–1460.

(63) Frisch, M. J. et al. Gaussian 16 Revision C.01. 2016; Gaussian Inc. Wallingford CT.

(64) Stewart, J. J. P. MOPAC2009. Stewart Computational Chemistry, Colorado Springs, CO, USA, http://OpenMOPAC.net (2008).

(65) Ołdziej, S.; Kozłowska, U.; Liwo, A.; Scheraga, H. A. Determination of the Potentials of Mean Force for Rotation About $C^{\alpha} \cdots C^{\alpha}$ Virtual Bonds in Polypeptides from the *Ab Initio* Energy Surfaces of Terminally-Blocked Glycine, Alanine, and Proline. *J. Phys. Chem. A* **2003**, *107*, 8035–8046.

(66) Sieradzan, A. K.; Hansmann, U. H. E.; Scheraga, H. A.; Liwo, A. Extension of UNRES Force Field to Treat Polypeptide Chains with D-Amino Acid Residues. *J. Chem. Theory Comput.* **2012**, *8*, 4746–4757.

(67) Takahashi, O.; Kobayashi, K.; Oda, A. Computational Insight into the Mechanism of Serine Residue Racemization. *Chem. Biodivers.* **2010**, *7*, 1625–1629.

(68) Gijs Schaftenaar, Molden. `https://www3.cmbi.umcn.nl/molden/`.
